# Bcl10-controlled Malt1 paracaspase activity is key for the immune suppressive function of regulatory T cells

**DOI:** 10.1038/s41467-019-10203-2

**Published:** 2019-05-28

**Authors:** Marc Rosenbaum, Andreas Gewies, Konstanze Pechloff, Christoph Heuser, Thomas Engleitner, Torben Gehring, Lara Hartjes, Sabrina Krebs, Daniel Krappmann, Mark Kriegsmann, Wilko Weichert, Roland Rad, Christian Kurts, Jürgen Ruland

**Affiliations:** 1 Institute of Clinical Chemistry and Pathobiochemistry, TUM School of Medicine, Technical University of Munich, 81675 Munich, Germany; 2 TranslaTUM, Center for Translational Cancer Research, Technical University of Munich, 81675 Munich, Germany; 3 German Cancer Consortium (DKTK), 69120 Heidelberg, Germany; 4 Research Unit Cellular Signal Integration, Helmholtz Zentrum München, German Research Center for Environmental Health, 85764 Neuherberg, Germany; 5 Institute of Experimental Immunology, Rheinische-Friedrichs-Wilhelms University of Bonn, 53127 Bonn, Germany; 6 School of Medicine, Institute of Virology, Technical University of Munich, 81675 Munich, Germany; 7 German Center for Infection Research (DZIF), Partner Site Munich, 81675 Munich, Germany; 8 Institute of Molecular Oncology and Functional Genomics, TUM School of Medicine, Technical University of Munich, 81675 Munich, Germany; 9 Institute of Pathology, University Hospital Heidelberg, 69120 Heidelberg, Germany; 10 Institute of Pathology, Technical University of Munich, 81675 Munich, Germany

**Keywords:** Lymphocyte activation, Regulatory T cells, NF-kappaB, Tumour immunology

## Abstract

Regulatory T cells (Tregs) have crucial functions in the inhibition of immune responses. Their development and suppressive functions are controlled by the T cell receptor (TCR), but the TCR signaling mechanisms that mediate these effects remain ill-defined. Here we show that CARD11-BCL10-MALT1 (CBM) signaling mediates TCR-induced NF-κB activation in Tregs and controls the conversion of resting Tregs to effector Tregs under homeostatic conditions. However, in inflammatory milieus, cytokines can bypass the CBM requirement for this differentiation step. By contrast, CBM signaling, in a MALT1 protease-dependent manner, is essential for mediating the suppressive function of Tregs. In malignant melanoma models, acute genetic blockade of BCL10 signaling selectively in Tregs or pharmacological MALT1 inhibition enhances anti-tumor immune responses. Together, our data uncover a segregation of Treg differentiation and suppressive function at the CBM complex level, and provide a rationale to explore MALT1 inhibitors for cancer immunotherapy.

## Abstract (web summary)

The differentiation and function of regulatory T (Treg) cells are critically controlled by T cell receptor (TCR) signaling. Here the authors show that CARD11-BCL10-MALT1 (CBM) complexes are dispensable for effector Treg conversion under inflammatory conditions but are critical for mediating Treg suppressive activity in a MALT1 paracaspase-dependent manner.

## Introduction

CD4^+^FoxP3^+^ regulatory T cells (Tregs) have crucial functions in the inhibition of immune responses^1^. An absence of Tregs due to genetic inactivation of their lineage defining transcription factor FoxP3^2,3^ or via induced Treg ablation after birth^4^ results in lethal auto-inflammatory syndromes with unrestricted activation of conventional T cells (Tconv) and other immune cells. However, Tregs are not only essential for the maintenance of immune homeostasis and the prevention of autoimmunity, but are also frequently enriched in tumors of various histologies^5^ where their inhibitory functions also restrict anti-tumor immunity and thereby promote malignant progression. Because of these protective and pathogenic Treg activities, there is a strong interest in understanding the mechanisms that control Treg differentiation and their suppressive effector function.

The early development of FoxP3^+^ Tregs in the thymus and periphery, the subsequent differentiation of naive resting Tregs (rTregs) to effector Tregs (eTregs), and their immune suppressive activities are all controlled by signals from the T cell antigen receptor (TCR)^6^. However, the TCR signaling pathways that differentially control Treg differentiation and suppressor functions remain insufficiently defined. Signaling complexes composed of CARD11, BCL10, and MALT1 (CBM complexes) mediate TCR-induced canonical activation of nuclear factor-κB (NF-κB) transcription factors in conventional T cells^7^, and recent studies highlighted crucial cell-intrinsic roles of canonical NF-κB signaling in Treg development and function for immune homeostasis^8–11^.

Upon TCR ligation and receptor proximal signaling, CBM complexes form large scaffolds around the central adapter molecule BCL10 and the associated paracaspase MALT1 that recruit ubiquitin regulators such as TRAF6 and UBC13^12^ to induce the activation of the IκB kinase (IKK). The activated catalytic subunit IKK2 then phosphorylates inhibitory IκB proteins to induce their proteolytic degradation, which allows the nuclear translocation of the canonical NF-κB subunits p65 and c-Rel to activate gene transcription^7^. However, while this pathway is essential for NF-κB activation after cognate antigen recognition, inflammatory cytokines can engage IKK2 via alternative signalosomes to activate NF-κB even in the absence of the CBM complex in T cells^13–16^. Moreover, within assembled CBM complexes, the paracaspase MALT1 has a dual role: it not only functions as scaffold for IKK2 activation, but it also exhibits proteolytic activity within a catalytic domain that tunes the NF-κB response by cleaving regulators such as A20, RelB, and CYLD^17^, and in addition inactivates the RNA-degrading proteins Regnase-1 and Roquin^18,19^, which control immune regulatory messenger RNA (mRNA) stability^20^. In contrast to the functions of the CBM complex in IKK regulation, the biological functions of the paracaspase activity in the immune system are much less understood.

Complete germline deficiencies of *CBM* in mice and also in humans result in combined immunodeficiencies, which are caused by severe defects in antigen-mediated conventional lymphocyte activation and a subsequent failure to induce protective adaptive immunity^7,21^. *CBM*-deficient mice as well as patients are also highly deficient in Foxp3^+^ Tregs^7,21^, which demonstrates that this pathway is also critical for the early development of precursors into the FoxP3^+^ Treg lineage. This early differentiation of Tregs requires the proteolytic activity of MALT1, as knock-in mice that only express protease-inactive MALT1 are also Treg deficient^22–24^ and develop an autoimmune disease that is rescued by the transfer of wild-type Treg cells^24^. Because of this early Treg differentiation block in the absence of an intact CBM complex, the functions and mechanisms of CBM signaling within mature Tregs—after these FoxP3^+^ cells have been established—is currently unknown.

To study the biological roles of CBM signaling in established Tregs, we create a series of conditional loss- and gain-of-function mutant mouse lines. We demonstrate that CBM signaling controls TCR-induced canonical NF-κB signaling in FoxP3^+^ Tregs, and drives the conversion of rTregs to eTregs under homeostatic conditions. However, in inflammatory milieus, cytokine signaling can bypass the requirement for the CBM complex for the rTreg to eTreg conversion. Nevertheless, within these cells, the CBM complex is absolutely critical for the Treg immune suppressive program. This Treg-suppressive activity is controlled by the MALT1 paracaspase activity in a non-redundant fashion, which mediates the upregulation of a set of Treg suppression markers including CTLA4. In proof-of-principle experiments, we also demonstrate that an acute blockade of CBM signaling within established Tregs or systemic pharmacological MALT1 paracaspase inhibition can enhance anti-tumor immunity in models of malignant melanoma. Thus, our study defines context-specific functions of the CBM complex for Treg differentiation and immune suppression, and provides preclinical evidence that may encourage the exploitation of MALT1 inhibitors in immune oncology.

## Results

### *Bcl10* in mature Tregs prevents autoimmune inflammation

To explore the cell-intrinsic functions of CBM signaling in Tregs, we first created a conditional *Bcl10* allele (*Bcl10*^*fl*^) (Supplementary Fig. 1a and b). For validation, we crossed *Bcl10*^*fl/fl*^ animals with CD4-Cre mice^25^. In *Bcl10*^*fl/fl*^;CD4-Cre offspring *Bcl10* is deleted at the double-positive stage of thymic T cell development, leading to BCL10 deficiency in peripheral T cells and severe reductions in the number of FoxP3^+^ Tregs (Supplementary Fig. 1c–e), demonstrating that the known essential functions for BCL10 signaling for early Treg development are T cell lineage intrinsic.

**Fig. 1:**
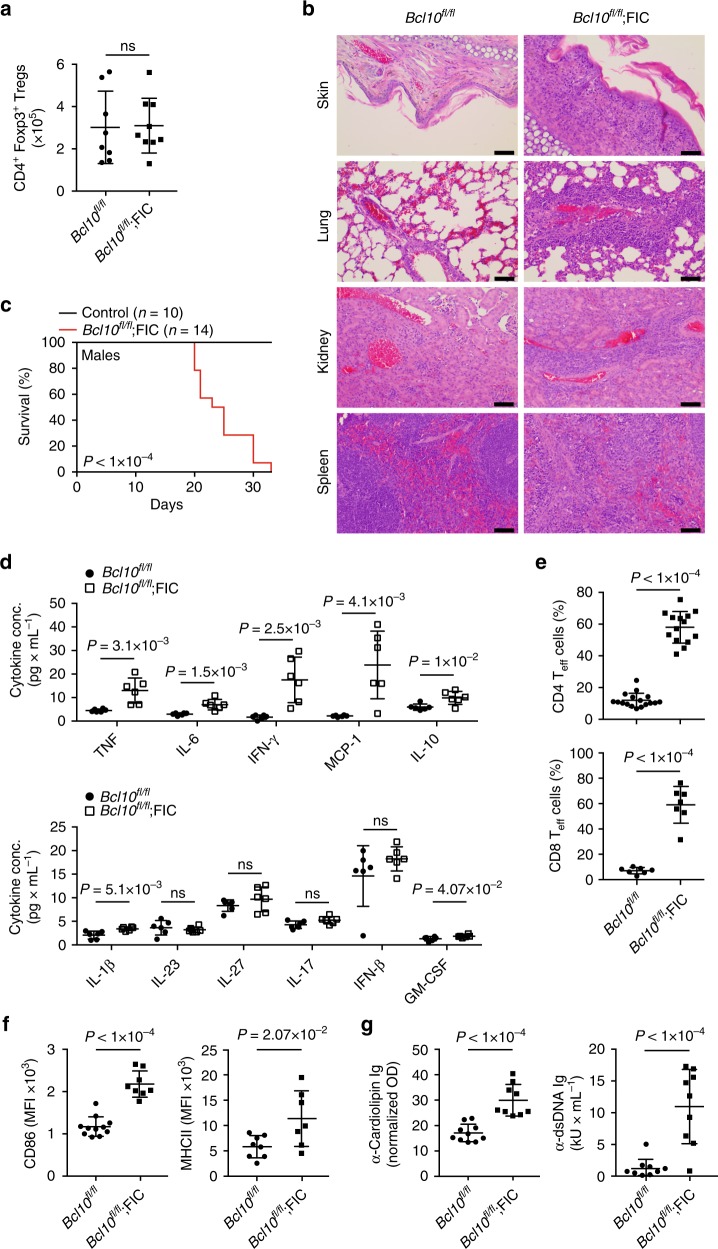
*Bcl10* disruption in mature regulatory T cells (Tregs) results in autoimmune inflammation. **a** Quantified analysis of the total numbers of viable splenic CD4^+^Foxp3^+^ Tregs of 16-day-old male *Bcl10*^*fl/fl*^;FIC or *Bcl10*^*fl/fl*^ control mice. Data are cumulative from four independent experiments. **b** Histological hematoxylin and eosin (H.E.) staining of the indicated organs on day 25 post-partum. The black bar in the lower right corner depicts the scale of 50 μm. Pictures are representative of ≥2 mice per genotype. **c** Survival curves of male *Bcl10*^*fl/fl*^ or *Bcl10*^*+/fl*^;FIC control mice (black line, *n* = 10) versus *Bcl10*^*fl/fl*^;FIC mice (red line, *n* = 14). Survival equals the day the animal had to be sacrificed to avoid severe burden. Statistical significance between the survival curves with the corresponding *P* value was calculated by a log-rank (Mantel–Cox) test. **d** Concentration of indicated inflammatory cytokines in the sera of 16-day-old male *Bcl10*^*fl/fl*^ (dots) and *Bcl10*^*fl/fl*^;FIC (squares) mice. Each dot or square represents one mouse. **e** Frequency of CD4^+^Foxp3^–^ (upper panel) and CD8^+^ (lower panel) CD44^hi^CD62L^lo^ effector T cells (T_eff_) in the spleens of 16-day-old male *Bcl10*^*fl/fl*^ and *Bcl10*^*fl/fl*^;FIC mice as measured by fluorescence-activated cell sorting (FACS). Data are cumulative from seven or three independent experiments. **f** Median fluorescence intensity (MFI) of cell surface CD86 (left) and MHCII (right) on CD19^+^B220^+^-gated splenic B cells of 16-day-old male *Bcl10*^*fl/fl*^ and *Bcl10*^*fl/fl*^;FIC mice. Data are cumulative from four or three independent experiments. **g** Quantification of anti-cardiolipin (left) and anti-dsDNA immunoglobulin (right) concentrations in the sera of 16-day-old male *Bcl10*^*fl/fl*^ and *Bcl10*^*fl/fl*^;FIC mice. The bars in **a** and **d**–**g** represent the mean ± SD; statistical significance between *Bcl10*^*fl/fl*^ and *Bcl10*^*fl/fl*^;FIC mice was assessed by a two-tailed unpaired Student’s *t* test. Significance values are depicted in the graph; (ns) not significant. Source data are provided as a Source Data File

To disrupt *Bcl10* within FoxP3^+^ Tregs after they have developed, we crossed *Bcl10*^*fl/fl*^ mice with *Foxp3*^*IRES-Cre*^ (FIC) animals^26^. Because the *Foxp3* locus is on the X-chromosome, male FIC mice express Cre in virtually all Treg cells^27^. Strikingly, although the total number of FoxP3^+^ Tregs does not differ between male *Bcl10*^*fl/fl*^;FIC mice and control animals (Fig. 1a), *Bcl10*^*fl/fl*^;FIC mice develop a rapidly progressing wasting syndrome with massive inflammatory cell infiltration and auto-inflammatory pathology in the skin, lung, kidney, and lymphoid organs, requiring euthanasia at approximately 24 days of age (Fig. 1b, c, Supplementary Fig. 2a). The disease is characterized by a systemic increase in inflammatory cytokines, including tumor necrosis factor (TNF), interleukin-1β (IL-1β), IL-6, interferon-γ (IFN-γ), monocyte chemotactic protein-1, IL-10, and granulocyte–macrophage colony-stimulating factor (Fig. 1d), a massive activation of Tconv cells with an accumulation of CD44^hi^CD62L^lo^CD4^+^ and CD44^hi^CD62L^lo^CD8^+^ effector lymphocytes (Fig. 1e and Supplementary Fig. 2b) and pathological B cell activation with auto-antibody production (Fig. 1f, g). This disease of *Bcl10*^*fl/fl*^;FIC mice resembles the *Scurfy* phenotype caused by a complete absence of Tregs with regard to onset, progression, and pathology^2–4^, demonstrating that BCL10 signaling within established Tregs is absolutely critical for the maintenance of immune homeostasis.

### BCL10 regulates the homeostatic rTreg to eTreg conversion

**Fig. 2:**
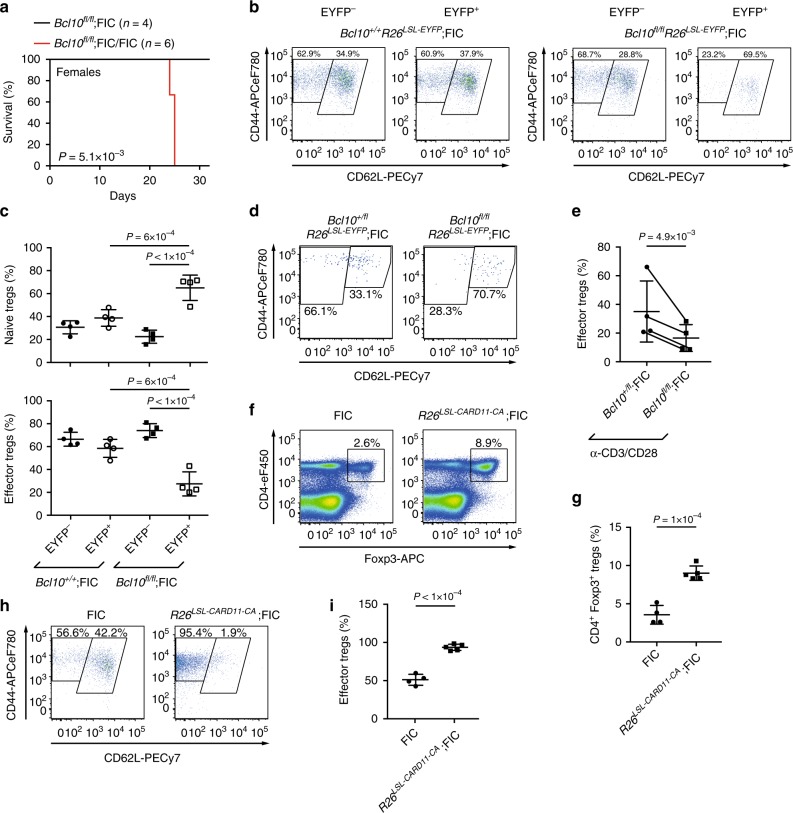
BCL10 signaling controls the homeostatic resting regulatory T cell (rTreg) to effector Treg (eTreg) conversion. **a** Survival curves of *Bcl10*^*fl/fl*^;FIC (black line, *n* = 4) versus *Bcl10*^*fl/fl*^;FIC/FIC female mice (red line, *n* = 6). *P* value was calculated by a log-rank (Mantel–Cox) test. **b** Fluorescence-activated cell sorting (FACS) profiles to detect either EYFP^–^ (left) or EYFP^+^ (right) CD62L^hi^ naive rTregs and CD44^hi^CD62L^lo^ eTregs in the viable CD4^+^Foxp3^+^ splenic Treg population of *Bcl10*^*+/+*^*Rosa26*^*LSL-EYFP*^;FIC (left plots) and *Bcl10*^*fl/fl*^*Rosa26*^*LSL-EYFP*^;FIC (right plots) female mice. Plots are representative of four mice each. **c** Frequencies of splenic EYFP^–^- and EYFP^+^-gated CD62L^hi^ naive rTregs (upper panel) and CD44^hi^CD62L^lo^ eTregs (lower panel) in the viable CD4^+^Foxp3^+^ Treg population of female *Bcl10*^*+/+*^;*Rosa26*^*LSL-EYFP*^;FIC and *Bcl10*^*fl/fl*^;*Rosa26*^*LSL-EYFP*^;FIC mice. Statistical significances were assessed by ordinary one-way analysis of variance (ANOVA) combined with Tukey’s multiple comparisons test. **d** Representative FACS experiment to detect the differentiation of sorted splenic CD4^+^EYFP^+^CD62L^hi^ naive rTregs of ≥2 pooled female *Bcl10*^*fl/fl*^;*Rosa26*^*LSL-EYFP*^;FIC and *Bcl10*^*+/fl*^;*Rosa26*^*LSL-EYFP*^;FIC control mice into CD4^+^EYFP^+^CD44^hi^CD62L^lo^ eTregs following 3 days of stimulation with anti-CD3/CD28. **e** Quantified percentages of differentiated eTregs. Data are from four independent experiments with ≥2 pooled mice per genotype. To avoid prominent effects of single data points on the mean, statistical significance was assessed by a two-tailed ratio-paired *t* test with corresponding paired data points of one experiment connected by a line. **f** FACS analysis to detect viable splenic CD4^+^Foxp3^+^ Tregs in 12-week-old FIC (*n* = 4) or *Rosa26*^*LSL-CARD11-CA*^;FIC female mice (*n* = 5). **g** Frequency of viable splenic CD4^+^Foxp3^+^ Tregs in 12-week-old FIC or *Rosa26*^*LSL-CARD11-CA*^;FIC female mice. **h** Representative FACS profile detecting the percentages of CD62L^hi^ rTregs and CD44^hi^CD62L^lo^ eTregs within the viable CD4^+^Foxp3^+^ splenic Treg population of 12-week-old FIC (*n* = 4) or *Rosa26*^*LSL-CARD11-CA*^;FIC (*n* = 5) female mice. **i** Frequencies of viable splenic CD44^hi^CD62L^lo^ eTregs in FIC control or *Rosa26*^*LSL-CARD11-CA*^;FIC female mice. To detect the transgene in **h**, **i**, gating was performed on CD4^+^Foxp3^+^GFP^+^ cells. Statistical significances in **g**, **i** were assessed by a two-tailed unpaired Student’s *t* test. Bars in **c**, **e**, **g**, **i** represent the mean ± SD. Data in **c** are representative of three independent experiments, while data in **f**–**i** are cumulative from two independent experiments. Source data are provided as a Source Data File

In female mice with one FIC allele, random X inactivation leads to Cre expression in only half of the Treg population^27^. Therefore, female *Bcl10*^*fl/fl*^;FIC mice are—in contrast to female *Bcl10*^*fl/fl*^; FIC/FIC mice with two FIC alleles—mosaic with BCL10-deficient and BCL10-expressing Tregs. Since 50% of the non-deleted Tregs are sufficient to maintain immune homeostasis (Fig. 2a), we can use these *Bcl10*^*fl/fl*^;FIC animals to study BCL10-deleted Tregs without the confounding effects of inflammation. To track the Cre-expressing cells, we introduced an EYFP reporter that is expressed from the *Rosa26* locus after Cre-mediated excision of a *loxP*-STOP-*loxP* (LSL) cassette (*Rosa26*^*LSL-EYFP*^)^28^. Under non-inflamed conditions, the frequencies of EYFP^+^ (as a surrogate for Cre-expressing *Bcl10*-deleted) rTregs, which are phenotypically identified by CD62L^hi^ rTregs surface marker expression, were increased approximately 3-fold in comparison to EYFP^*−*^ BCL10-expressing rTregs, while the frequencies of EYFP^+^CD44^hi^CD62L^lo^ surface marker expressing eTregs were 3-fold reduced (Fig. 2b, c). These data indicate a requirement of BCL10 for the rTreg to eTreg conversion, which depends on cognate antigen under homeostatic conditions^29,30^.

Next, we fluorescence-activated cell sorting (FACS) isolated CD4^+^EYFP^+^CD62L^hi^ rTregs from female *Bcl10*^*fl/fl*^*;Rosa26*^*LSL-EYFP*^;FIC or *Bcl10*^*+/fl*^*;Rosa26*^*LSL-EYFP*^;FIC control animals and stimulated these with anti-CD3 and anti-CD28 antibodies. Consistent with the in vivo data, BCL10-deficient rTregs are defective in the proper conversion into eTregs upon TCR/CD28 stimulation (Fig. 2d, e).

To genetically activate the BCL10 pathway selectively in Tregs in vivo, we next crossed FIC mice with *Rosa26*^*LSL-CARD11-CA*^ mice^31^ expressing a constitutively active CARD11 variant (CARD11^L225LI^, CARD11-CA) in Cre^+^ cells. We used this CARD11-CA mutant, originally isolated from a human lymphoma^32^, as a tool, as it autonomously enforces BCL10/MALT1 signaling in vivo^31^. CARD11-CA expression in Tregs resulted in a 2.5-fold increase in the frequency of Tregs in *Rosa26*^*LSL-CARD11-CA*^;FIC mice (Fig. 2f, g). This population is largely composed of eTregs (Fig. 2h, i), indicating that the selective activation of CBM signaling in Tregs in vivo is sufficient to promote rTreg to eTreg differentiation.

### Inflammation promotes *Bcl10*-independent eTreg conversion

**Fig. 3:**
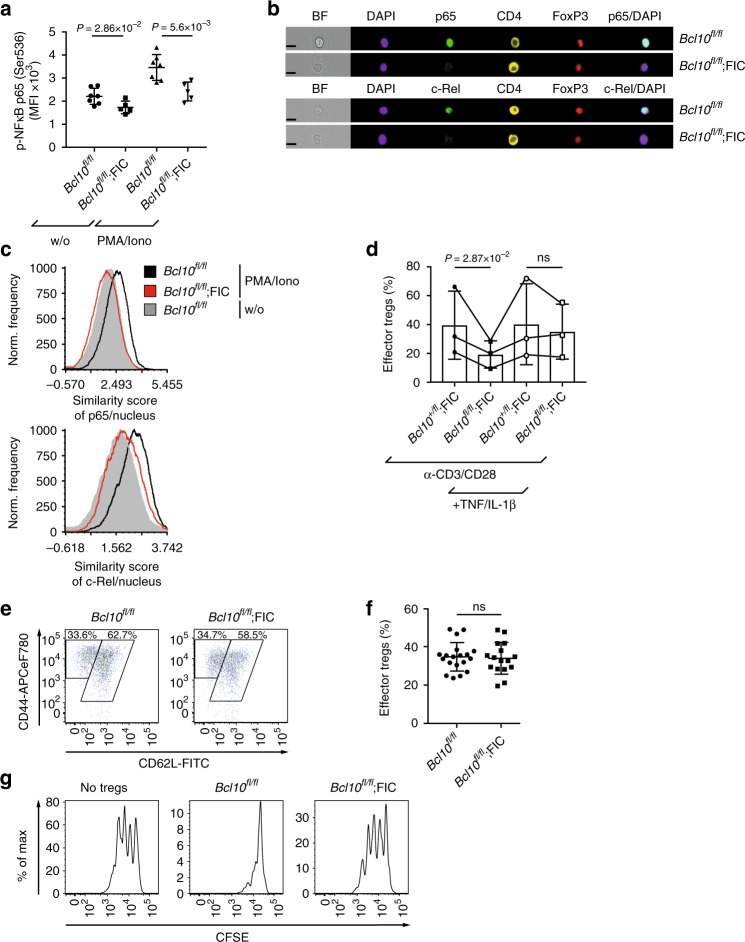
Inflammatory signals promote effector regulatory T cell (eTreg) conversion but not suppression. **a** PhosFlow to detect the median fluorescence intensity (MFI) of phospho-nuclear factor-κB (p-NF-κB) p65 at serine 536 in viable CD4^+^Foxp3^+^ splenic Tregs of 16-day-old male *Bcl10*^*fl/fl*^ and *Bcl10*^*fl/fl*^;FIC mice without (w/o) or with phorbol myristate acetate (PMA)/ionomycin. **b** Image stream analysis to detect the nuclear translocation of the NF-κB subunits p65 (upper two images) and c-Rel (lower two images) in CD4^+^Foxp3^+^-gated splenic Tregs of 16-day-old male *Bcl10*^*fl/fl*^ or *Bcl10*^*fl/fl*^;FIC mice upon PMA/ionomycin. 4′,6-Diamidino-2-phenylindole (DAPI) was used as a nuclear stain. Black bars represent 10 μm. (BF) brightfield. **c** Histogram profiles of the similarity scores of p65 (upper panel) or c-Rel (lower panel) and the DAPI stain. Black (*Bcl10*^*fl/fl*^) and red (*Bcl10*^*fl/fl*^;FIC) histograms represent the similarity score in splenic CD4^+^Foxp3^+^ Tregs upon PMA/ionomycin. The gray histogram indicates unstimulated splenic CD4^+^Foxp3^+^ Tregs (*Bcl10*^*fl/fl*^). **d** Quantified analysis of the differentiation of sorted splenic CD4^+^EYFP^+^CD62L^hi^ rTregs of ≥2 pooled female *Bcl10*^*fl/fl*^;*Rosa26*^*LSL-EYFP*^;FIC and *Bcl10*^*+/fl*^;*Rosa26*^*LSL-EYFP*^;FIC mice into CD4^+^EYFP^+^CD44^hi^CD62L^lo^ eTregs in the absence or presence of tumor necrosis factor (TNF (20 ng mL^*−*1^) and interleukin-1β (IL-1β) (20 ng mL^*−*1^) and anti-CD3/CD28. Statistical significance was assessed by a ratio-paired *t* test; paired data points of one experiment are connected by a line. Data are cumulative of three independent experiments and illustrate three points without cytokines of Fig. 2e again; (ns) not significant. **e** Fluorescence-activated cell sorting (FACS) profile detecting splenic CD62L^hi^ rTregs and CD44^hi^CD62L^lo^ eTregs in the viable CD4^+^Foxp3^+^ cell gate of 16-day-old male *Bcl10*^*fl/fl*^ and *Bcl10*^*fl/fl*^;FIC mice. **f** Frequencies of splenic CD44^hi^CD62L^lo^ eTregs in the viable CD4^+^Foxp3^+^ cell gate of 16-day-old male *Bcl10*^*fl/fl*^ or *Bcl10*^*fl/fl*^;FIC mice. **g** In vitro Treg-suppressor assay: carboxyfluorescein succinimidyl ester (CFSE)-labeled naive conventional CD4^+^ T cells were cultivated at a 2:1 ratio without Tregs (left plot), with sorted CD4^+^CD25^+^CD45RB^lo^ Tregs of 16-day-old male *Bcl10*^*fl/fl*^ (middle plot) or *Bcl10*^*fl/fl*^;FIC (right plot) mice in the presence of irradiated splenocytes and anti-CD3. FACS plots are representative of five independent experiments and show the proliferation of viable CFSE^+^-gated cells. Statistical significances in **a** and **f** were calculated by a two-tailed unpaired Student’s *t* test. Bars in **a**, **d**, and **f** represent the mean ± SD. Data are representative of **b**, **c** two or **e** eight independent experiments and cumulative from **a** two or **f** eight independent experiments. Source data are provided as a Source Data File

Because the conversion of rTreg to eTreg requires both TCR signaling^29,30^ and NF-κB activation^9^, we next stimulated *Bcl10*-deficient Tregs with phorbol myristate acetate/ionomycin (PMA/ionomycin) as a pharmacological mimic of TCR signaling and determined NF-κB activation by intracellular FACS analysis. In line with the known function of BCL10 in conventional T cells^13^, *Bcl10*-deficient Tregs showed a significant reduction in PMA/ionomycin-induced p65 phosphorylation (Fig. 3a). Moreover, quantitative imaging flow cytometric cell analysis revealed defective nuclear translocation of the canonical NF-κB subunits p65 and c-Rel (Fig. 3b, c).

Canonical NF-κB signaling in T cells is also activated by inflammatory cytokines. As indicated above, factors such as TNF or IL-1β can bypass the requirement of BCL10 for NF-κB activation and engage the IKK complex in a CBM-independent manner^13–15^. Therefore, we tested whether the addition of inflammatory cytokines could rescue the rTregs to eTregs conversion defect in TCR-stimulated *Bcl10*-deficient Tregs. Indeed, supplementing TNF and IL-1β in vitro promoted the conversion of *Bcl10*-deficient rTregs to the eTreg phenotype in the presence of a TCR stimulus (Fig. 3d and Supplementary Fig. 3a).

Male *scurfy*-like *Bcl10*^*fl/fl*^; FIC mice also exhibit high inflammatory cytokine concentrations in vivo (Fig. 1d). In these inflamed environments, we also observed normal frequencies of *Bcl10*-deficient eTregs that retain their phenotypic markers in vitro (Fig. 3e, f and Supplementary Fig. 3b). Together with the data above, this indicates that inflammatory signals can overcome the BCL10 dependency for the rTreg to eTreg conversion in vitro and in vivo. However, these *Bcl10*-deficient Tregs are defective in their suppressive activity in in vitro suppression assays (Fig. 3g and Supplementary Fig. S3c), which is consistent with the lethal inflammatory disease in male *Bcl10*^*fl/fl*^;FIC mice.

**Fig. 4:**
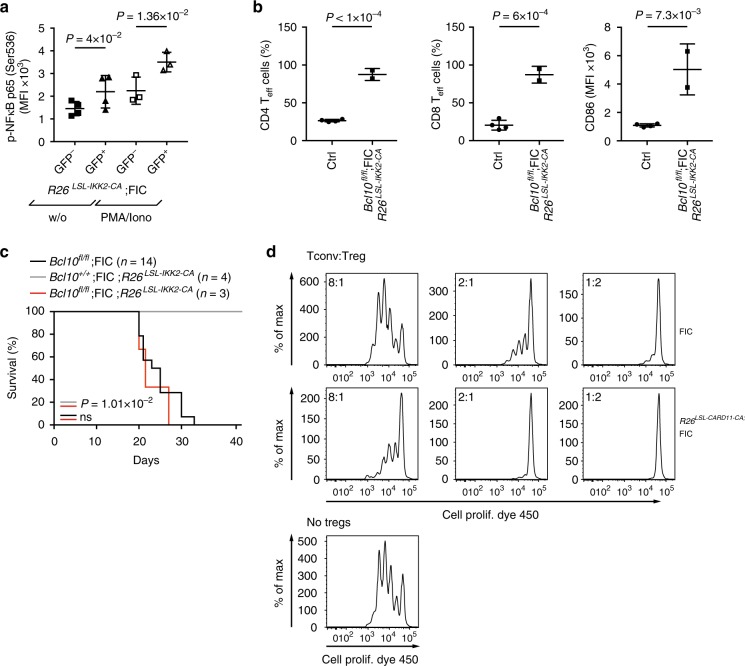
Enforced IκB kinase (IKK) activation cannot rescue the *Bcl10* deficiency in regulatory T cells (Tregs). **a** PhosFlow experiment to detect the median fluorescence intensity (MFI) of phospho-nuclear factor-κB (p-NF-κB) p65 at serine 536 in the viable CD4^+^Foxp3^+^ Treg population in spleen samples of *Rosa26*^*LSL-IKK2-CA*^;FIC female mice with or without (w/o) PMA/ionomycin stimulation. Due to random X inactivation, female mice were mosaic with GFP^+^ Tregs expressing the IKK2-CA variant and GFP^–^ non-IKK2-CA-expressing Tregs. Statistical significances between the GFP^–^ and GFP^+^ populations were assessed by a two-tailed paired *t* test. **b** Quantified analysis of CD4^+^Foxp3^–^CD44^hi^CD62L^lo^-gated splenic CD4 T effector (T_eff_) cells (left graph), CD8^+^CD44^hi^CD62L^lo^-gated splenic CD8 T_eff_ cells (middle graph), and the median fluorescence intensity (MFI) of cell surface CD86 on CD19^+^B220^+^-gated splenic B cells (right graph) of either wild-type control (Ctrl) or diseased *Bcl10*^*fl/fl*^Rosa26^*LSL-IKK2-CA*^;FIC male mice. A statistical significance between genotypes was assessed by a two-tailed unpaired Student’s *t* test. **c** Survival curves of *Bcl10*^*+/+*^*Rosa26*^*LSL-IKK2-CA*^;FIC control (gray line, *n* = 4), *Bcl10*^*fl/fl*^*Rosa26*^*LSL-IKK2-CA*^;FIC (red line, *n* = 3), and *Bcl10*^*fl/fl*^;FIC male mice (black line, *n* = 14). Survival equals the day the animal had to be sacrificed to avoid severe burden. Statistical significances between the survival curves were calculated by a log-rank (Mantel–Cox) test; (ns) not significant. **d** In vitro Treg-suppressor assay assessing the suppressor activity of Tregs of FIC and *Rosa26*^*LSL-CARD11-CA*^;FIC mice. Sorted splenic CD4^+^CD25^–^CD45RB^hi^ naive conventional T cells of FIC mice were labeled with Cell Proliferation Dye eFluor 450 and cultivated for 3 days without any Tregs (lower panel), with sorted splenic CD4^+^CD25^+^CD45RB^lo^ Tregs of FIC mice (upper panel) or with sorted splenic CD4^+^CD25^+^CD45RB^lo^GFP^+^ Tregs of *Rosa26*^*LSL-CARD11-CA*^;FIC mice (middle panel) in the presence of irradiated splenocytes and soluble anti-CD3. FACS plots show the proliferation profiles of Cell Prolif Dye 450^+^-gated cells cultivated with different ratios of Tregs. Bars in **a**, **b** represent the mean ± D. Data in **a**, **b** are cumulative from two independent experiments and data in **d** are representative of three independent experiments. Source data are provided as a Source Data File

To activate canonical NF-κB signaling genetically in *Bcl10*-deficient Tregs, we enforced the pathway by crossing *Bcl10*^*fl/fl*^;FIC mice with *Rosa26*^*LSL-IKK2-CA*^ mice, which conditionally express a constitutively active IKK2 version (IKK2-CA) from the *Rosa26* locus^33^. Flow cytometric analysis confirmed that IKK2-CA constitutively activates NF-κB signaling in Tregs from *Rosa26*^*LSL-IKK2-CA*^;FIC mice (Fig. 4a). Nevertheless, neither the autoimmune inflammatory phenotype nor the overall survival of male *Bcl10*^*fl/fl*^;FIC mice was rescued in *Bcl10*^*fl/fl*^;*Rosa26*^*LSL-IKK2-CA*^;FIC mice (Fig. 4b, c). Thus, BCL10 signaling in addition mediates the activation of the Treg-suppressive program in a manner that is not rescued by enforced canonical IKK2 activity. In line with a Treg suppression program that is triggered by CBM signaling, sorted Treg populations from *Rosa26*^*LSL-CARD11-CA*^;FIC mice with enforced CBM signaling are indeed hyperactive in in vitro suppression assays (Fig. 4d).

### MALT1 protease activity controls Treg suppression

**Fig. 5:**
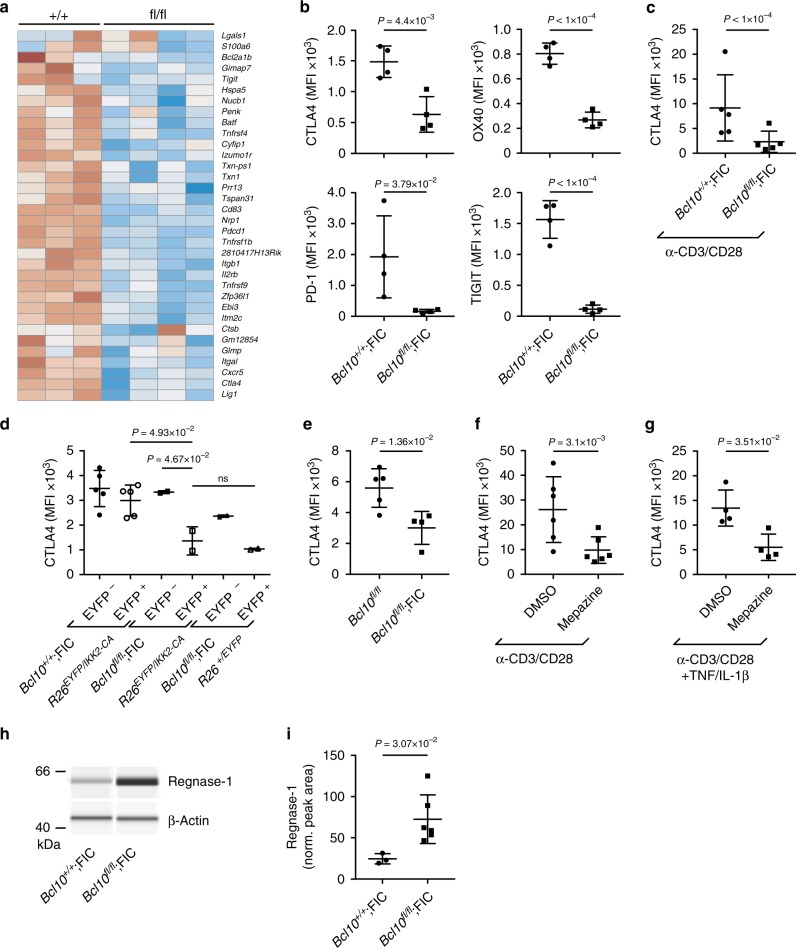
BCL10 and MALT1 proteolytic activity control regulatory T cell (Treg) suppression. **a** Heat map of a subcluster of differentially expressed genes in sorted CD4^+^EYFP^+^CD44^hi^CD62L^lo^ splenic effector Tregs (eTregs) of *Bcl10*^*+/+*^*;Rosa26*^*LSL-EYFP*^;FIC (+/+) and *Bcl10*^*fl/fl*^*;Rosa26*^*LSL-EYFP*^;FIC (fl/fl) female mice. The blue color indicates a low abundance of messenger RNAs (mRNAs), whereas the red color depicts high mRNA expression. **b** Quantified median fluorescence intensities (MFIs) of CTLA4, OX40, PD-1, and TIGIT on splenic CD4^+^Foxp3^+^CD44^hi^CD62L^lo^-gated eTregs in *Bcl10*^*+/+*^;*Rosa26*^*LSL-EYFP*^;FIC and *Bcl10*^*fl/fl*^;*Rosa26*^*LSL-EYFP*^;FIC female mice. **c** MFI of CTLA4 in sorted CD4^+^EYFP^+^CD44^lo^CD62L^hi^ resting Tregs (rTregs) of ≥2 pooled female *Bcl10*^*fl/fl*^*;Rosa26*^*LSL-EYFP*^;FIC and *Bcl10*^*+/fl*^*;Rosa26*^*LSL-EYFP*^;FIC control mice following 3 days of differentiation with anti-CD3/CD28. Data are cumulative from four independent experiments; statistical significance was assessed by a two-tailed ratio-paired *t* test. **d** MFI of CTLA4 on viable EYFP^–^ or EYFP^+^ CD4^+^Foxp3^+^CD44^hi^CD62L^lo^-gated eTregs of female *Bcl10*^*+/+*^*;Rosa26*^*LSL-EYFP/LSL-IKK2-CA*^;FIC controls, *Bcl10*^*fl/fl*^*;Rosa26*^*LSL-EYFP/LSL-IKK2-CA*^;FIC and *Bcl10*^*fl/fl*^*;Rosa26*^*LSL-EYFP*^;FIC mice. Statistical significances were calculated by ordinary one-way analysis of variance (ANOVA) combined with Tukey’s multiple comparisons test; (ns) not significant. **e** MFIs of CTLA4 on viable splenic CD4^+^Foxp3^+^CD44^hi^CD62L^lo^-gated eTregs in 16-day-old *Bcl10*^*fl/fl*^ and *Bcl10*^*fl/fl*^;FIC mice. Data are cumulative from two independent experiments with ≥2 mice each. **f**, **g** CTLA4 expression on in vitro differentiated, sorted CD4^+^EYFP^+^CD44^lo^CD62L^hi^ rTregs of ≥2 pooled female *Bcl10*^*+/+*^*;Rosa26*^*LSL-EYFP*^;FIC or *Bcl10*^*+/fl*^*;Rosa26*^*LSL-EYFP*^;FIC control mice after anti-CD3/CD28 stimulation in the presence of the MALT1 protease inhibitor mepazine (5 μM) **f** alone or **g** with added tumor necrosis factor (TNF (20 ng mL^*−*1^) and interleukin-1β (IL-1β (20 ng mL^*−*1^). Dimethyl sulfoxide (DMSO) was used as a control. The graphs show the MFI of viable CD4^+^EYFP^+^-gated cells on day 3 from **f** six and **g** two independent experiments. Statistical significance was assessed by a two-tailed ratio-paired *t* test. **h** Western immunoassay (WES) immunoblot analysis of Regnase-1 expression in sorted CD4^+^EYFP^+^ Tregs of male *Bcl10*^*fl/fl*^;FIC or *Bcl10*^*+/+*^;FIC control mice. β-Actin served as a loading control. The bands shown are from one WES immunoblotting run. **i** Normalized Regnase-1 to β-actin peak area of different biological replicates analyzed for Regnase-1 and β-actin expression using the WES immunoblotting system. Each dot represents one mouse. Statistical significances (**b**, **e**, **i**) were assessed by a two-tailed unpaired Student’s *t* test. Bars in **b**–**g** and in **i** represent the mean ± SD. Source data are provided as a Source Data File

To identify the CBM-controlled suppressive Treg programs that are required to maintain immune homeostasis, we next FACS-isolated CD4^+^EYFP^+^CD44^hi^CD62L^lo^ eTreg cells from non-inflamed female *Bcl10*^*fl/fl*^*;Rosa26*^*LSL-EYFP*^;FIC and control animals and performed RNA sequencing. Subsequent comparative analysis of variance (ANOVA)-based analysis revealed a failure of *Bcl10*-deficient eTregs to express a functionally relevant signature of the eTreg suppressive phenotype, which includes the set of mRNAs encoding CTLA4, OX40, PD-1, and TIGIT^8,11,29^ (Fig. 5a). Subsequent flow cytometry data further demonstrated that *Bcl10*-deficient eTregs fail to regularly express these selected receptors on the surface, which are cooperatively involved in Treg-mediated immune suppression (Fig. 5b and Supplementary Fig. 4a).

Since no single effector molecule accounts for the entire spectrum of the Treg-suppressive program^34^, we next focused our mechanistic analysis on the CBM-dependent regulation of CTLA4 expression, as a prototypic marker molecule of the Treg-suppressive phenotype^26^. To this end, we first stimulated FACS-isolated naive rTregs from female *Bcl10*^*fl/fl*^*;Rosa26*^*LSL-EYFP*^;FIC mice via their TCR. While *Bcl10*^*+/fl*^*;Rosa26*^*LSL-EYFP*^;FIC control Tregs upregulated CTLA4 as expected, *Bcl10*-deficient rTregs are severely impaired in CTLA4 upregulation (Fig. 5c). This failure of TCR-induced CTLA4 expression was not rescued by enforced NF-κB activation via IKK2-CA in BCL10-deficient eTregs from non-inflamed *Bcl10*^*fl/fl*^*;Rosa26*^*LSL-IKK2-CA*^;FIC female mice (Fig. 5d). Moreover, *Bcl10*-deficient eTregs from the inflamed environments of *scurfy*-like male *Bcl10*^*fl/fl*^;FIC mice were also defective in CTLA4 expression (Fig. 5e). Thus, inflammatory cytokines or the selective activation of NF-κB are not sufficient to confer regular CTLA4 expression in the absence of BCL10. Conversely, the enforced activation of CBM signaling within Tregs from *Rosa26*^*LSL-CARD11-CA*^;FIC mice resulted in a markedly enhanced CTLA4 expression (Supplementary Fig. 4b).

The expression of CTLA4 is not only controlled by gene transcription, but in particular tightly regulated at the level of the *Ctla4* mRNA, which is continuously degraded by Regnase-1^19^. Because the MALT1 protease can cleave and inactivate Regnase-1 in conventional T lymphocytes to stabilize *Ctla4* mRNA for CTLA4 upregulation^19^, we tested whether an analogous pathway operates in Tregs. Therefore, we first pharmacologically inhibited the MALT1 paracaspase in sorted wild-type rTregs. Indeed, MALT1 inhibition prevented TCR-induced CTLA4 upregulation (Fig. 5f). Moreover, the addition of TNF and IL-1β was unable to confer regular CTLA4 upregulation in the presence of MALT1 inhibitors (Fig. 5g). In addition, FACS-isolated Tregs from diseased *Bcl10*^*fl/fl*^;FIC mice exhibited massively enhanced Regnase-1 protein levels (Fig. 5h, i), indicating that they fail to degrade this ribonuclease.

### MALT1 protease activity mediates Treg suppression in vivo

Because the data above indicate that the MALT1 proteolytic activity might control the Treg-suppressive program, we created further mouse models to test this hypothesis genetically. Previously, we and others had engineered a MALT1 paracaspase mutant (PM) knock-in strain with an inactivating point mutation in the MALT1 protease domain^22–24,35^. In these animals, the catalytically inactive MALT1-PM variant is expressed at endogenous levels and retains the ability to assemble CBM scaffolds for IKK-mediated NF-κB activation^22–24,35^. For this study, we generated in addition a conditional *Malt1*^*fl/fl*^ strain for cell type-specific ablation of a *floxed Malt1* wild-type allele, which we first intercrossed with FIC mice^26^ to generate *Malt1*^*fl/fl*^;FIC mice. Male *Malt1*^*fl/fl*^;FIC or female *Malt1*^*fl/fl*^;FIC/FIC mice develop a lethal auto-inflammatory syndrome with a massive pathological activation of Tconv and B cells (Supplementary Fig. 5a-f) with the same kinetics and severity compared to *Bcl10*^*fl/fl*^;FIC mice (Fig. 1a–g) or FoxP3-deficient *scurfy* mice^2^. In the inflamed environments of diseased male *Malt1*^*fl/fl*^;FIC and female *Malt1*^*fl/fl*^;FIC/FIC animals, the frequency of eTregs was unaltered (Supplementary Fig. 5g). However, under non-inflamed conditions in female *Malt1*^*fl/fl*^;FIC mice (that harbor a *Rosa26*^*LSL-EYFP*^ reporter allele for the identification of the *Malt1*-deleted EYFP^+^ cells in the mosaic environment), the frequencies of EYFP^+^ eTregs were approximately 2-fold reduced in comparison to EYFP^−^ MALT1-expressing eTregs (Supplementary Fig. 6a). In addition, under both homeostatic and inflammatory conditions, *Malt1*-deficient eTregs fail to regularly express CTLA4 (Supplementary Fig. 6b and c). Moreover, similar to *Bcl10*-deficient Tregs (Fig. 3g), *Malt1*-deficient Tregs are defective in their suppressive activity in in vitro suppression assays (Supplementary Fig. 6d). These data demonstrate that *Malt1* is like *Bcl10* essential for the rTreg to eTreg conversion and for the suppressive capacity of Tregs.

**Fig. 6:**
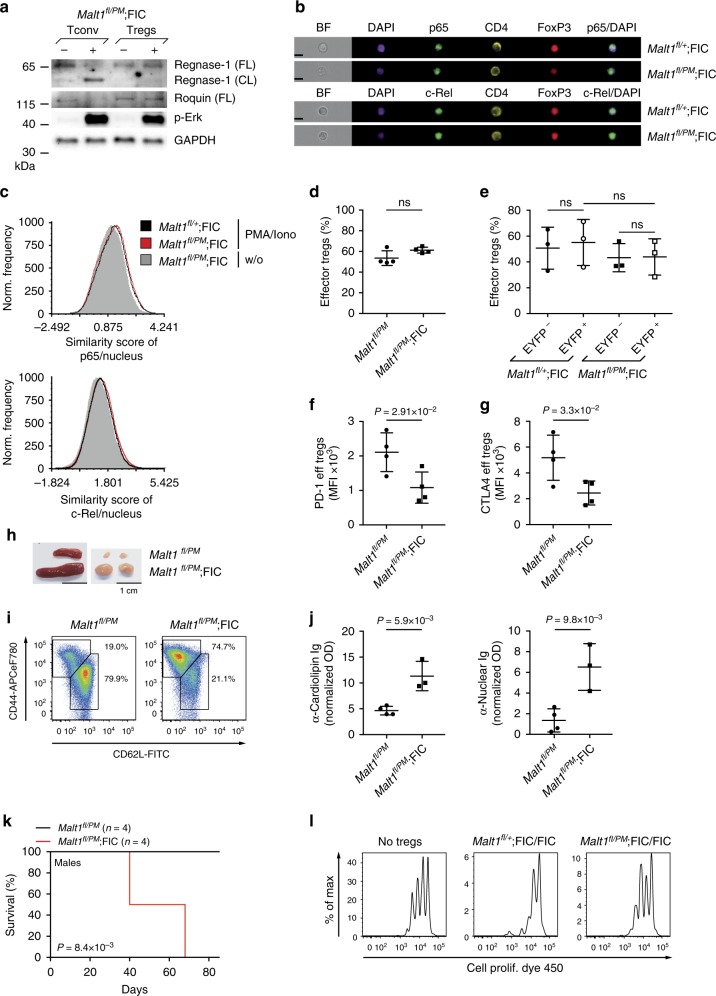
MALT1 protease activity in regulatory T cells (Tregs) is critical for immune suppression. **a** Immunoblot analysis to detect full-length (FL) and cleaved (CL) Regnase-1, and FL Roquin in sorted CD4^+^EYFP^–^CD25^–^CD45RB^hi^ naive conventional T cells (Tconv) and CD4^+^EYFP^+^ Tregs of diseased male *Malt1*^*fl/PM*^;*Rosa26*^*LSL-EYFP*^;FIC mice minus (−) or plus (+) phorbol myristate acetate (PMA)/ionomycin. Phospho (p)-Erk served as a stimulation control, and glyceraldehyde 3-phosphate dehydrogenase (GAPDH) as a loading control. **b** Image stream analysis detecting nuclear translocation of p65 (upper two images) and c-Rel (lower two images) in splenic CD4^+^Foxp3^+^ Tregs of male *Malt1*^*fl/PM*^;FIC and *Malt1*^*fl/+*^;FIC mice upon PMA/ionomycin. 4′,6-Diamidino-2-phenylindole (DAPI) was used as a nuclear stain. Black bars indicate 10 μm. (BF) brightfield. **c** Histograms of the similarity score of p65 (upper panel) or c-Rel (lower panel) and the DAPI stain. Black (*Malt1*^*fl/+*^;FIC) and red (*Malt1*^*fl/PM*^;FIC) histograms represent the similarity score in splenic CD4^+^Foxp3^+^ Tregs upon PMA/ionomycin. The gray histogram indicates unstimulated splenic CD4^+^Foxp3^+^ Tregs (*Malt1*^*fl/PM*^;FIC). **d** Frequencies of splenic CD44^hi^CD62L^lo^ effector Tregs in the viable CD4^+^Foxp3^+^ cell gate of male *Malt1*^*fl/PM*^ and *Malt1*^*fl/PM*^;FIC mice **e** Frequencies of EYFP^–^- and EYFP^+^-gated CD44^hi^CD62L^lo^ effector Tregs in the viable CD4^+^Foxp3^+^ cell gate of female *Malt1*^*fl/+*^;*Rosa26*^*LSL-EYFP*^;FIC and *Malt1*^*fl/PM*^;*Rosa26*^*LSL-EYFP*^;FIC mice. Statistical significances were assessed by ordinary one-way analysis of variance (ANOVA) combined with Tukey’s multiple comparisons test. **f**, **g** Quantified median fluorescence intensities (MFIs) of **f** PD-1 and **g** CTLA4 on viable CD4^+^Foxp3^+^CD44^hi^CD62L^lo^-gated splenic effector (eff) Tregs in male *Malt1*^*fl/PM*^;FIC and *Malt1*^*fl/PM*^ mice. **h** Size of spleens (left) and lymph nodes (right) in male *Malt1*^*fl/PM*^;FIC (*n* = 4) and *Malt1*^*fl/PM*^ (*n* = 4) mice, aged 40 days. Scale bars represent 1 cm. **i** FACS plots of CD44^hi^CD62L^lo^CD4^+^Foxp3^–^-gated splenic cells of *Malt1*^*fl/PM*^ (*n* = 4) and *Malt1*^*fl/PM*^;FIC (*n* = 4) mice. **j** Quantified serum concentrations of anti-cardiolipin (left) and anti-nuclear immunoglobulin (right) in male *Malt1*^*fl/PM*^;FIC and *Malt1*^*fl/PM*^ mice. **k** Survival curves of male *Malt1*^*fl/PM*^;FIC (red line, *n* = 4) and *Malt1*^*fl/PM*^ control (black line, *n* = 4) mice. Statistical significance was calculated by a log-rank (Mantel–Cox) test. **l** In vitro Treg-suppressor assay with sorted and Cell Proliferation Dye 450-labeled naive Tconv cells cultivated at a 2:1 ratio without any Tregs (left panel), with sorted splenic CD4^+^EYFP^+^ Tregs of either *Malt1*^*fl/+*^;*Rosa26*^*LSL-EYFP*^;FIC/FIC (middle panel) or *Malt1*^*fl/PM*^;*Rosa26*^*LSL-EYFP*^;FIC/FIC mice (right panel) in the presence of irradiated splenocytes and anti-CD3. FACS plots show the proliferation of viable cells. Bars in **d**–**g** and **j** represent the mean ± SD. Data are representative of **a**–**c**, **l** two or cumulative from **d**–**g** two independent experiments with **d**, **f**, **g** *n* = 2 or **b**, **c**, **l** *n* = 3 mice per genotype. Statistical significances in **d**, **f**, **g**, **j** were assessed by a two-tailed unpaired Student’s *t* test. Source data are provided as a Source Data File

To specifically explore the enzymatic function MALT1 in Tregs, we next crossed *Malt1*^*fl/fl*^ animals to *Malt1*^*PM*/*+*^ mice to obtain *Malt1*^*fl/PM*^ animals, which were viable, healthy, and without overt immunological defects, demonstrating that the proteolytic activity from one *Malt1*^*fl*^ allele is sufficient to maintain homeostasis (Fig. 6k). Then, we intercrossed *Malt1*^*fl/PM*^ animals with FIC mice^26^ to create *Malt1*^*fl/PM*^;FIC mice, in which the protease-competent *Malt1*^*fl*^ allele is specifically deleted in FoxP3^+^ cells and which allows us to selectively study the biological function of the MALT1 protease activity within Tregs in vivo without considering its scaffold role.

In contrast to isolated conventional T cells from *Malt1*^*fl/PM*^;FIC mice, FACS-sorted Tregs from *Malt1*^*fl/PM*^;FIC mice are defective in Regnase-1 and Roquin cleavage upon stimulation with PMA/ionomycin (Fig. 6a), confirming defective MALT1 protease activity specifically in Tregs. Yet, paracaspase-mutated Tregs from *Malt1*^*fl/PM*^;FIC mice maintain—as expected from the intact MALT1 scaffold function—their ability to activate the NF-κB subunits p65 and c-Rel upon PMA/ionomycin stimulation (Fig. 6b, c, Supplementary Fig. 7a)—in contrast to *Bcl10*-deficient Tregs (Fig. 3b). In vivo, *Malt1*^*fl/PM*^;FIC mice exhibit regular frequencies of rTregs and eTregs populations (Fig. 6d), even in non-diseased female *Malt1*^*fl/PM*^;FIC mice (Fig. 6e). Importantly, however, the expression of the eTreg-suppressive markers PD-1 and CTLA4 is abrogated in paracaspase-mutated Tregs (Fig. 6f, g and Supplementary Fig. 7b), demonstrating that their expression is critically controlled by the MALT1 protease activity. Strikingly, *Malt1*^*fl/PM*^;FIC mice also develop a spontaneous and lethal *scurfy*-like phenotype with splenomegaly and lymphadenopathy, auto-inflammatory pathology with an activation and accumulation of CD44^hi^CD62L^lo^CD4^+^ and CD44^hi^CD62L^lo^CD8^+^ conventional effector T lymphocytes, and pathological B cell activation with auto-antibody production (Fig. 6h–k, Supplementary Fig. 7c and d). Consistent with this auto-inflammatory disease, sorted Tregs of *Malt1*^*fl/PM*^;FIC mice are defective in their suppressive capacity in vitro (Fig. 6l), demonstrating that the MALT1 proteolytic activity within Tregs is absolutely essential for their suppressor function in vitro and for the suppression of cellular and humoral immunity in vivo, and this activity cannot be compensated for in inflammatory environments.

### Inhibition of CBM signaling can enhance anti-tumor immunity

**Fig. 7:**
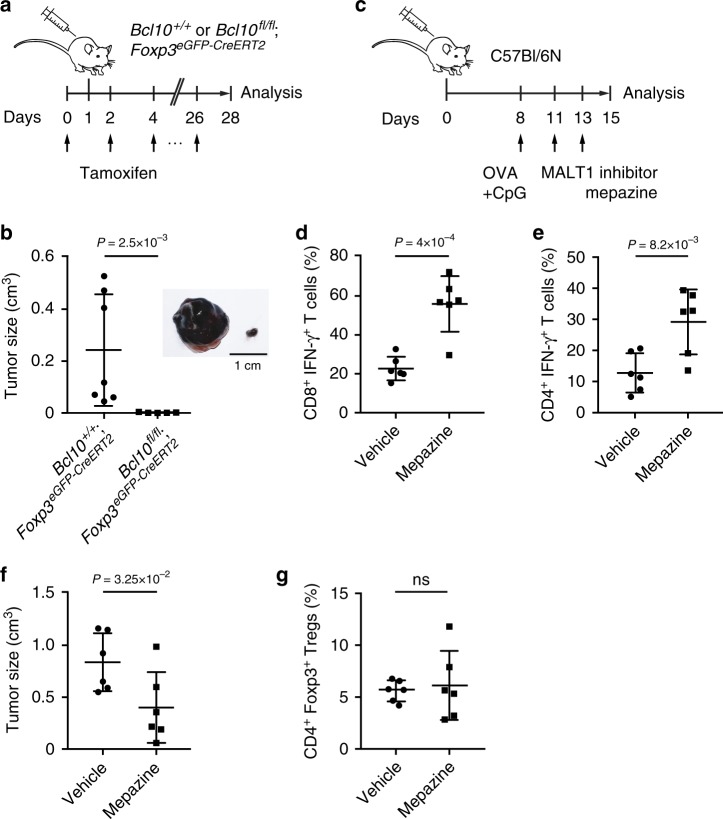
Inhibition of CARD11-BCL10-MALT1 (CBM) signaling enhances anti-tumor immunity. **a** Schematic representation of the B16F1 tumor model combined with the acute deletion of *Bcl10* in regulatory T cells (Tregs): on day 1, 1 × 10^5^ B16F1 tumor cells were subcutaneously injected into the flanks of *Bcl10*^+/+^;*Foxp3*^*eGFP-CreERT2*^ and *Bcl10*^*fl/fl*^;*Foxp3*^*eGFP-CreERT2*^ mice. Tamoxifen was administered every other day deleting the *Bcl10*^*fl/fl*^ alleles in newly emerging Tregs until the final analysis on day 28. **b** Quantification of the tumor size in *Bcl10*^+/+^;*Foxp3*^*eGFP-CreERT2*^ and *Bcl10*^*fl/fl*^;*Foxp3*^*eGFP-CreERT2*^ mice on day 28 after tamoxifen treatment. Representative tumors of *Bcl10*^+/+^;*Foxp3*^*eGFP-CreERT2*^ (left) and *Bcl10*^*fl/fl*^;*Foxp3*^*eGFP-CreERT2*^ (right) male mice are depicted on the right side of the graph. Scale bar represents 1 cm. Tumor size was calculated as follows: *V* = (*L* × *W*^2^) × *2*^*−*1^, where *L* is the length of tumor (mm) and *W* the width of tumor (mm). Statistical significance between genotypes was assessed by a two-tailed Mann–Whitney *U* test. **c** Schematic representation of the B16-OVA tumor model in wild-type mice combined with a pharmacological inhibition of the MALT1 protease activity: B16-OVA cells were subcutaneously injected into the flanks of C57Bl/6  mice, followed by a vaccination with OVA + CpG on day 8. On days 11 and 13, mice were treated with either the MALT1 inhibitor mepazine or vehicle (phosphate-buffered saline (PBS)/5% dimethyl sulfoxide (DMSO)) and analyzed on day 15. **d**, **e** Quantification of the ratio of tumor-infiltrating **d** CD8^+^IFN-γ^+^ cells to the frequency of CD8^+^ cells and **e** CD4^+^Foxp3^*−*^IFN-γ^+^ cells to the total percentage of CD4^+^Foxp3^*−*^ T cells after re-stimulation of enriched tumor-infiltrating lymphocytes with phorbol myristate acetate (PMA) (100 nM)/ionomycin (1 µM). Statistical significance was calculated with a two-tailed unpaired Student’s *t* test. **f** Tumor size on day 15 following treatment on days 11 and 13 with either vehicle or mepazine (16 mg kg^*−*1^ bodyweight). Statistical significance between groups was assessed with a one-tailed Mann–Whitney *U* test. **g** Quantification of the ratio of tumor-infiltrating Tregs to the total percentage of T cells. A statistical difference of the mean was calculated by a two-tailed unpaired Student’s *t* test; (ns) not significant. Bars in **b**, **d**–**g** indicate the mean ± SD. IFN-γ interferon-γ. Source data are provided as a Source Data File

Because of the biomedical interest in developing strategies that can block Treg function in tumor microenvironments, we finally performed proof-of-principle experiments and tested whether an inhibition of CBM signaling in Tregs could enhance anti-tumor immunity. We choose a model of melanoma, because Tregs inhibit anti-tumor immune responses in these cancers, where their presence correlates with inferior clinical outcome^36^. To acutely manipulate CBM signaling in Tregs in a syngeneic model of melanoma in vivo, we crossed *Bcl10*^*fl/fl*^ mice with *Foxp3*^*eGFP-CreERT2*^ animals^37^, which express a tamoxifen-inducible Cre recombinase in FoxP3^+^ cells. Subsequently, we used adult *Bcl10*^*fl/fl*^;*Foxp3*^*eGFP-CreERT2*^ offspring with a homeostatic immune system and acutely deleted *Bcl10* in mature Tregs by tamoxifen treatment (Fig. 7a). The acute blockage of CBM signaling in Tregs released a strong anti-tumor immune response, leading to a massive reduction in the final melanoma size (Fig. 7b), highlighting that continuous CBM signaling within Tregs maintains their suppressive capacity in tumor microenvironments. Furthermore, the induced deletion of *Bcl10* in Tregs results in systemic immune activation, demonstrating that the immune inhibitory effects of BCL10 signaling within Tregs are independent of defects in Treg development (Supplementary Fig. 8a–d). Next, we established a melanoma model in wild-type mice and pharmacologically blocked MALT1 paracaspase activity in these animals in vivo (Fig. 7c). The systemic MALT1 inhibition also enhanced anti-tumor immunity in a vaccination protocol with an increase in the activity of tumor-infiltrating IFN-γ-producing CD8^+^ and CD4^+^ T cells and a significant decrease in tumor size, although this treatment did not alter the frequency of Tregs within the tumor tissue (Fig. 7d–g, Supplementary Fig. 9a and b). Moreover, in tumor bearing *Malt1*-deficient mice, mepazine did not affect the growth of the MALT1-competent tumor cells themselves (Supplementary Fig. 9c and d).

## Discussion

In conclusion, our genetic in vivo analysis demonstrates key functions of CBM signaling within the mature Treg lineage. The CBM signalosome controls TCR-induced NF-κB activation, mediates the rTreg to eTreg conversion under non-inflamed conditions, and is in particular critical for the Treg-suppressive activity through a mechanism that extensively depends on the MALT1 protease function.

The critical cell-intrinsic roles of NF-κB signaling in Treg differentiation and function have recently been established^8–11^. Genetic deficiencies of IKK2, RelA, and c-Rel or the combined deficiencies of RelA and c-Rel in Tregs revealed unexpected cell type-specific functions of the canonical NF-κB pathway in the development of Tregs and the maintenance of the Treg-suppressive phenotype during homeostasis and in tumor microenvironments. These studies initiated promising efforts to exploit NF-κB signaling for tumor immunotherapy, for example, by utilizing IKK2 or NF-κB c-Rel inhibitors^8–11^. We demonstrate that the CBM complex controls Treg-intrinsic TCR-mediated activation of NF-κB—comparable to its function in Tconv cells—and mediates rTreg to eTreg conversion in homeostasis. Nevertheless, BCL10-deficient Tregs are in contrast to conditionally IKK2, RelA- or c-Rel-deleted Tregs^8–11^ not NF-κB or IKK deficient, and inflammatory cytokines can activate NF-κB in a CBM-independent manner^13–15^. Consistently, TNF and IL-1β can promote rTreg to eTreg conversion even in the absence of BCL10, and we detected normal numbers of eTregs in inflamed tissues of male *scurfy*-like *Bcl10*^*fl/fl*^;FIC mice. Nevertheless, these *Bcl10*-deficient Tregs as well as *Malt1*-deficient Tregs are entirely defective in providing immune suppression, as the disease in *Bcl10*^*fl/fl*^;FIC mice or *Malt1*^*fl/fl*^;FIC mice phenocopies the severe *Scurfy* disease of animals with complete Treg deficiency with regard to kinetics and severity^2–4^. In addition, whereas constitutive active IKK2 expression can rescue the Treg suppression defect in *Ubc13*^*fl/fl*^;Foxp3-GFP-hcre mice^38^, which lack the ubiquitin-conjugating enzyme UBC13 in Tregs that specifically connects the CBM complex to NF-κB activation, the genetic enforcement of NF-κB activity via IKK2-CA expression entirely failed to rescue the immune-suppressive defect of *Bcl10*-deficient Treg cells. Thus, these genetic and functional data altogether indicate that—although the CBM complex controls the Treg phenotype in part via TCR-mediated NF-κB signaling—it regulates additional IKK-independent immune-suppressive mechanisms.

Using further unequivocal genetic models, we pinpoint that the proteolytic function of MALT1 within mature Tregs is key for their immune-suppressive function. While the MALT1 paracaspase activity is not required to assemble the CBM scaffolds for PMA/ionomycin-mediated RelA and c-Rel nuclear translocation, the paracaspase cleaves and inactivates the RNA-degrading enzymes Regnase-1 and Roquins in activated Tregs. These factors can post-transcriptionally control large sets of immune regulatory mRNAs including *Ctla4*^18,19^. We find that BCL10-deficient or MALT1 protease-inhibited Tregs fail to degrade Regnase-1 and Roquin and fail to upregulate CTLA4—which we use as a marker of the Treg immune-suppressive phenotype—and multiple additional factors—which cooperatively mediate the still insufficiently characterized Treg-suppressive program. Moreover, while previous studies using in vitro generated MALT1-deficient iTregs^39^ have reported that these cells can show activity in in vitro suppressive assays, our conditional in vivo mutagenesis demonstrates a non-redundant function of the paracaspase in Treg-suppressive activity in vivo. Therefore, our data indicate a two-step model: first, the CBM complex activates the canonical NF-κB pathway to initiate the NF-κB-mediated Treg programs and second, it controls via the MALT1 proteolytic activity an additional layer of the Treg-suppressive phenotype, which cannot be compensated in inflammatory environments. This MALT1 protease-regulated program presumably depends on the control of mRNA stability and could in addition involve MALT1 substrates such as A20, RelB, and CYLD, which execute secondary modulatory effects on the NF-κB pathway^17^, which needs to be investigated in future studies. In addition, the BCL10-MALT1 scaffold likely controls further paracaspase-independent factors that mediate Treg suppression, as the autoimmune inflammatory disease in *Malt1*^*fl/PM*^;FIC mice develops with slightly delayed kinetics in comparison to the disease of *Bcl10*^*fl/fl*^;FIC mice or *Malt1*^*fl*/*fl*^;FIC animals.

To develop future Treg-based therapies that can enhance effector immunity by Tconv cells, it is important to uncover the mechanisms that are critical for the function of one cell type, but dispensable for the other. We observed that the CBM complex as a unit controls not only the well-established activation of conventional effector T cells, but intriguingly also the opposing suppressor activity in Tregs. Consistently, the acute deletion of BCL10 only in Tregs can release protective anti-tumor immune responses. More importantly, however, we also highlight that the MALT1 protease activity is key for the Treg-suppressor activity, while previous studies from us and independent groups have found that the paracaspase activity is to a considerable extent dispensable for the activation of conventional T cells^22–24,35^. This second fact is dramatically underscored by the spontaneous destructive lymphocyte-mediated autoimmunity in homozygous *Malt1*^*PM/PM*^ paracaspase mutant mice. Thus, the autoreactive CD4^+^ and CD8^+^ paracaspase-mutated Tconv cells that develop in Treg-deficient *Malt1*^*PM/PM*^ mice—but not in *Malt1*^*−/−*^ mice^40^—do not depend on MALT1 proteolytic activity for effector function^22–24,35^. Together with our new findings, these data provide evidence that the proteolytic function of MALT1 operates as a switch in the immune system that segregates the Treg-suppressor pathways from conventional T cell activation.

Several small-molecule MALT1 inhibitors are currently under development for potential clinical applications^41–43^. Initially, these programs were started to develop compounds for lymphoma therapy and triggered by the observation that the paracaspase activity mediates survival of certain human lymphoma cells^44,45^. Our new study provides now a rationale to also test these compounds in cancer immunotherapy settings. In initial experiments, we observe that a pharmacological inhibition of the paracaspase can enhance anti-tumor immune responses in vivo. In line with our cell type-specific genetic analysis, it is likely that much of the beneficial consequences of MALT1 inhibitors in the tested melanoma model are mediated via inhibitory effects on Tregs. Nevertheless, the systemic treatment with MALT1 inhibitors will also affect the paracaspase activity in conventional T cells, antigen-presenting cells, and other tumor microenvironmental cells. Therefore, our here presented study should prompt further systematic explorations that dissect the contributions of MALT1 paracaspase function in individual cell types in complex tumor microenvironments. Moreover, Tregs represent only one mechanism that tumor tissues utilize to subvert the elimination of cancer cells by the immune system. Our identification of the MALT1 paracaspase as a rational target for cancer immunotherapy should also be investigated in combination with other modalities, including cytotoxic drugs that induce immunogenic cell death and immune checkpoint inhibitors.

## Methods

### Mice

All mouse experiments were carried out in accordance with the guidelines of the Federation of European Laboratory Animal Science Association (FELASA) and followed the legal approval of the Government of Upper Bavaria (Regierung von Oberbayern). We complied with all relevant ethical regulations for animal testing, research, and euthanasia by cervical dislocation.

The *Bcl10*^+/*fl*;*neo*^ allele was generated by flanking exon 2 of *Bcl10* with *loxP* sites. More specifically, the targeting construct contained a 0.7 kb short arm (SA) of *Bcl10* intron 1, a *frt*-flanked neomycin resistance cassette followed by a 1.3 kb *loxP*-flanked conditional arm comprising exon 2 and a 5 kb long arm, including exon 3 of the *Bcl10* locus. Following linearization and electroporation of the vector into R1/E ES cells, successful homologous recombination was screened for by PCR and confirmed by standard Southern blot analysis using 5′ and 3′ probes. Positive clones were then injected into C57BL/6 mice (R. Naumann, MPI-CBG, Dresden, Germany). Chimeric mice were mated for germline transmission of the targeted *Bcl10* allele and crossed with ACT-FLPe mice^46^ to delete the neomycin resistance cassette. *Bcl10*^*fl*^ mice were generated as a mixed 129X1x129S1xC57BL/6J background and crossed for at least four generations onto C57BL/6N background.

CD4-Cre transgenic mice^25^, *Foxp3*^*eGFP-CreERT2*^ mice^37^, and *Rosa26*^*LSL-EYFP*^ mice^28^ were ordered from Jackson Laboratory and were on the genetic background indicated by the Jackson Laboratory. *Rosa26*^*LSL-IKK2-CA*^ mice^33^ on a C57BL/6N background were provided by M. Schmidt-Supprian (Technical University of Munich). *Foxp3*^*IRES-Cre*^ (FIC) mice^26^, *Rosa26*^*LSL-CARD11-CA*^^31^ mice, and the *Malt1*^*PM*^ strain^22^ were on C57BL/6N background and have been described. *Malt1*^*fl*^ mice were generated by blastocyst injection of the Malt1^tm1a/EUCOMM)Hmgu^ (C57BL/6N-A/a) ES cell clone HEPD0618_3_D10 from the EuMMCR repository archive of mutant ES cells (German Research Center for Environmental Health, Helmholtz Center Munich, Munich, Germany). All mice were bred and maintained at the institute’s animal care facility under specific pathogen-free conditions and genotyped using the appropriate primers (Supplementary Table 1). The sex and age of the used mice is indicated in the figure legends; if not stated, adult mice aged 6–12 weeks were used for all experiments; littermate controls were used whenever possible.

### Cells

B16F1 cells were obtained from ATCC and cultured in Dulbecco’s modified Eagle’s medium (Thermo Fisher Scientific) supplemented with 10% (v v^−1^) fetal calf serum (FCS) (Capricorn Scientific) and 1% (v v^−1^) penicillin–streptomycin–glutamine (Thermo Fisher Scientific), while B16-OVA cells^10,47^ were grown in RPMI-1640 (Thermo Fisher Scientific) supplemented with 10% (v v^−1^) FCS (PAN-Biotech), 1% (v v^−1^) penicillin–streptomycin–glutamine (Thermo Fisher Scientific), and 0.4 mg mL^−1^ G418. Both cell lines were routinely tested for mycoplasma infection.

### Histology

Organs were formalin fixed, paraffin embedded, and cut (2 μm) before tissue sections were stained with hematoxylin and eosin. Images were acquired using either an Olympus BX53 microscope and CellSens Dimension software or an AxioVert (Zeiss) microscope with an AxioCam and processed by AxioVision software (Carl Zeiss).

### Sample preparation

Lymphoid organs were meshed, and erythrocytes were lysed using G-DEXIIb RBC Lysis Buffer (iNtRON Biotechnology). For enrichment of tumor-infiltrating lymphocytes (TILs), tumor samples were digested for 30 min at 37 °C with 2 mg mL^−1^ collagenase D (Roche) and 50 μg mL^−1^ DNase I (Roche) in RPMI-1640 (Thermo Fisher Scientific) supplemented with 10% (v v^−1^) FCS (Capricorn Scientific), 1% (v v^−1^) penicillin–streptomycin–glutamine (Thermo Fisher Scientific), 1 mM sodium pyruvate (Thermo Fisher Scientific), 10 mM HEPES (Thermo Fisher Scientific), 1× Gibco MEM NE-AA (Thermo Fisher Scientific) and 56 µM β-mercaptoethanol (Thermo Fisher Scientific). Following the addition of a final concentration of 10 mM EDTA to the cell media, the cells were meshed, and TILs were enriched at the interface using a 36%/80% Percoll (GE Healthcare) gradient.

### Flow cytometry

Cells were washed once with phosphate-buffered saline (PBS), and live/dead cell staining was performed using a fixable viability dye diluted 1:1000 (eBioscience). After the cells were blocked with anti-CD16/32 clone 93 1:300 (eBioscience), they were stained with PBS/2% FCS with the following fluorochrome-coupled antibodies purchased from either eBioscience, BD Pharmingen or BioLegend: anti-B220 clone RA3-6B2 1:400, anti-CD4 clone GK1.5 1:400, anti-CD8 clone 53-6.7 1:400, anti-CD19 clone 1D3 1:400, anti-CD25 clone PC61.5 1:400, anti-CD44 clone IM7 1:400, anti-CD45RB C363.16A 1:400, anti-CD62L clone MEL.14 1:400, anti-CD86 clone GL1 1:400, anti-CTLA4 (CD152) clone UC10-4B9 1:300, anti-Foxp3 clone FJK-16s 1:300, anti-IFN-γ clone XMG1.2 1:300, anti-MHCII clone M5/114.15.2 1:400, anti-OX40 (CD134) clone OX86 1:300, anti-PD-1 (CD279) clone J43 1:300, and anti-TIGIT clone GIGD7 1:300. For sorting, antibodies were all diluted 1:200. For intracellular FACS, the cells were fixed with 2% formalin for 40 min, permeabilized by two washes with 1× perm/wash buffer (eBioscience), and stained overnight with the fluorochrome-coupled antibodies in 1× perm/wash buffer. For PhosFlow, splenocytes were either left untreated or stimulated with 100 nM PMA (Sigma) and 1 µM ionomycin (Merck) for 30 min at 37 °C and 5% CO_2_. Live/dead cell staining and anti-CD4 clone GK1.5 surface staining were performed as indicated, followed by intracellular FACS with anti-Foxp3 clone FJK-16s 1:300 (eBioscience) and anti-phospho-NFκB p65 (Ser536) 1:100 (93H1, Cell Signaling) antibodies. After cells were washed twice with perm/wash buffer, they were stained with an allophycocyanin-labeled anti-rabbit IgG antibody 1:1000 (A10931, Invitrogen). For cytokine staining, the cells were also stimulated with 100 nM PMA (Sigma) and 1 µM ionomycin (Merck) for 4 h at 37 °C and 5% CO_2_ in the presence of 1× brefeldin A (BioLegend). After the cells had undergone live/dead cell and surface staining, they were fixed and permeabilized using the Foxp3 staining kit (eBioscience). All cells were subjected to flow cytometric analysis on a FACSCanto II (BD Biosciences) or sorted with a FACS Aria III (BD Biosciences). Gating strategies to sort or analyze the respective cell population are depicted in Supplementary Fig. 10. FACS data were finally analyzed with FlowJo version 9.7.7 or 10.1r7.

### Cytokine bead array

Inflammatory cytokines were measured in mouse sera using the LEGENDplex Mouse Inflammation Panel (BioLegend) according to the manufacturer’s protocol.

### Enzyme-linked immunosorbent assay

Immunoglobulins against auto-antigens in mouse sera were measured using the respective auto-antibody ELISA (enzyme-linked immunosorbent assay) kits (Alpha Diagnostic International) according to the manufacturer’s instructions.

### In vitro Treg-suppressor assay

According to Collison and Vignali^48^, CD4^+^CD25^–^CD45RB^hi^ conventional naive T cells and CD4^+^CD25^+^CD45RB^lo^ Tregs were sorted with a FACS Aria III (BD Biosciences); in case of *Rosa26*^*LSL-EYFP*^;FIC reporter mice, CD4^+^EYFP^+^-gated Tregs were. Conventional CD4^+^ T cells were either labeled with 10 µM carboxyfluorescein succinimidyl ester (CFSE) (Sigma) or 10 µM Cell Proliferation Dye eFluor 450 (Thermo Fisher Scientific) according to the manufacturer’s protocol. Subsequently, 1.25 × 10^4^ Tregs were plated with labeled conventional naive CD4^+^ T cells in a 2:1, 1:1, and further two-fold ratios in RPMI-1640 (Thermo Fisher Scientific) supplemented with 10% (v v^–1^) FCS (Capricorn Scientific), 1% (v v^−1^) penicillin–streptomycin–glutamine (Thermo Fisher Scientific), 1 mM sodium pyruvate (Thermo Fisher Scientific), 10 mM HEPES (Thermo Fisher Scientific), 1× Gibco MEM NE-AA (Thermo Fisher Scientific) and 56 µM β-mercaptoethanol (Thermo Fisher Scientific) into a 96-well U-bottom plate. Following the addition of 1 µg mL^−1^ (final concentration) of soluble anti-CD3 clone 145-2C11 (BD Pharmingen) and 5 × 10^4^ splenocytes that had been irradiated with 30Gy using a Gulmay X-ray generator, the cells were incubated for 3 days at 37 °C and 5% CO_2_. Subsequently, the cells were subjected to flow cytometric analysis on a FACSCanto II (BD Biosciences).

### In vivo tamoxifen treatment and tumor experiments

*Bcl10*^*+/+* or *fl/fl*^;*Foxp3*^*eGFP-Cre-ERT2*^ hemizygous male mice and *Bcl10*^*+/+* or *fl/fl*^;*Foxp3*^*eGFP-CreERT2*^*/Foxp3*^*eGFP-CreERT2*^ female mice (6–10 weeks old) were treated perorally on day 0 with 5 mg tamoxifen (Hexal) in ClinOleic 20% (Baxter). On day 1, 1 × 10^5^ B16F1 tumor cells were subcutaneously injected into the left flanks of the mice, and subsequently, a peroral administration of 5 mg tamoxifen was repeated each second day to delete the floxed alleles in newly emerging Tregs until the final analysis on day 28. To assess the tumor growth under the pharmacological inhibition of MALT1 protease activity in vivo, 7-week-old C57BL/6N mice were subcutaneously injected on day 0 with 4 × 10^5^ B16-OVA cells into the flank. On day 8, mice subcutaneously received 50 mg of ovalbumin (Sigma-Aldrich) plus 10 nmol CpG 1668 (TIB Molbiol Berlin), and from day 11 onwards, 16 mg kg^−1^ bodyweight mepazine in PBS/5% dimethyl sulfoxide was intraperitoneally administered every other day until the final analysis on day 15. All tumor experiments received the ethical/legal approval by the Government of Upper Bavaria. We adhered to the maximum size of tumors (1500 mm^3^) requiring immediate termination of the experiment. Tumor size was measured at least every third day by an electronic caliber and calculated as follows: *V* = (*L* × *W*^2^) × 2^−1^, where *L* is the length of tumor (mm) and *W* the width of tumor (mm). Further, mice were assessed by their general behavior, behavior upon provocation, outer appearance, weight, respiration, and body condition (BC) parameters. Besides tumor volume, early termination also occurred after exulceration of the tumor, respiratory problems, an exceeded BC score (BC1), weight loss ≥20%, or apathy.

### Treg differentiation assay

CD4^+^EYFP^+^CD44^lo^CD62L^hi^-naIve rTregs of *Rosa26*^*LSL-EYFP*^;FIC female mice were sorted on a FACS Aria III (BD Biosciences) and plated in RPMI-1640 (Thermo Fisher Scientific) supplemented with 10% (v v^−1^) FCS (Capricorn Scientific), 1% (v v^−1^) penicillin–streptomycin–glutamine (Thermo Fisher Scientific), 1 mM sodium pyruvate (Thermo Fisher Scientific), 10 mM HEPES (Thermo Fisher Scientific), 1× Gibco MEM NE-AA (Thermo Fisher Scientific), and 56 µM β-mercaptoethanol (Thermo Fisher Scientific) into a 96-well U-bottom plate. After the MALT1 protease inhibitor mepazine (5 µM, Millipore) with or without 20 ng mL^−1^ TNF (Peprotech) and 20 ng mL^−1^ IL-1β (Peprotech) were added to the cells, they were stimulated with 4 µL/8000 cells Dynabeads Mouse T-Activator CD3/CD28 (Thermo Fisher Scientific) for 3 days at 37 °C and 5% CO_2_. Subsequently, the cells were stained and subjected to flow cytometric analysis on a FACSCanto II (BD Biosciences).

### Image stream analysis

Splenocytes were either left untreated or stimulated with 100 nM PMA (Sigma) and 1 µM ionomycin (Calbiochem) for 30 min at 37 °C, 5% CO_2_. Following a blocking step with anti-CD16/32 clone 93 (1:200) (eBioscience), cells were stained with fluorochrome-coupled anti-CD4 clone GK1.5 (1:200) and anti-CD25 clone PC61.clone (1:200) (eBioscience), and then subjected to intracellular staining with fluorochrome-coupled anti-Foxp3 clone FJK-16s (1:200) (eBioscience), anti-NFκΒ p65 sc-372 (1:200), or anti-c-Rel sc-71 (1:200) (both Santa Cruz Biotechnology) using the Foxp3 Staining Buffer Set (eBioscience) according to the manufacturer’s protocol. After the cells were stained with a Fluorescein isothiocyanate-coupled anti-rabbit IgG (1:200) (554020, BD Pharmingen) secondary antibody, 0.1 ng mL^−1^ 4′,6-diamidino-2-phenylindole (Thermo Fischer) was added as a nuclear stain. The stained cell suspensions were then subjected to flow cytometric analysis or an ImageStreamX Mark II (Amnis Merck Millipore). The data were analyzed using the IDEAS software (Amnis Merck Millipore) in which nuclear translocation is determined by a similarity score that quantifies the correlation of nuclear stain and translocation probe intensities. High correlation, and thus high similarity scores, represent strong nuclear translocation, whereas low scores are indicative of cytoplasmic localization. Histogram overlays were generated using FCSExpress6Plus (De Novo Software, Glendale, USA).

### Immunoblot

For detection of full-length and cleaved MALT1 substrates, cells were pre-incubated with 5 µM MG132 (ApexBio), and then stimulated for 90 min with 100 nM PMA (Sigma) and 1 µM ionomycin (Merck) at 37 °C, 5% CO_2_ and subjected to immunoblot analysis. For this purpose, cells were washed once with PBS and resuspended in RIPA buffer (50 mM Tris/Cl, pH 8.0, 150 mM NaCl, 1.0% (v v^−1^) NP-40, 0.5% (w v^−1^) deoxycholate) supplemented with protease inhibitors, 10 mM NaF and 4 mM Na_3_VO_4_. Following a 5 min incubation step on ice, the cell lysate was centrifuged at 20,000 × *g* at 4 °C. Subsequently, the supernatant was mixed with Laemmli buffer and boiled for 10 min at 95 °C. Denatured proteins were separated on a 10% polyacrylamide gel, transferred onto a nitrocellulose membrane (Whatman), and detected by the respective primary and secondary antibodies: anti-BCL10 clone C78F1 (1:1000) (Cell Signaling), anti-β-actin clone 8H10D10 (1:1000) (Cell Signaling), anti-Regnase-1 clone 604421 (1 µg mL^−1^) (R&D Systems), anti-Roquin clone 3F12^49^ (1:10), anti-p-Erk clone E10 (1:1000) (Cell Signaling), anti-GAPDH clone 6C5 (1:1000) (Calbiochem), anti-rabbit IgG-HRP (1:3000) (Cell Signaling), anti-mouse IgG-HRP (1:3000) (Cell Signaling), and anti-rat IgG-HRP (1:3000) (GE Healthcare). For the detection of low amounts of protein, Western immunoassay (ProteinSimple) was used according to the manufacturer’s protocol with anti-Regnase-1 clone 604421 (1:50) (R&D Systems) and a polyclonal anti-β-actin antibody (1:100) (#4967, Cell Signaling).

### RNA sequencing

In total, 1000 CD4^+^EYFP^+^CD44^hi^CD62L^lo^ effector eTregs from *Bcl10*^*+/+*^*;Rosa26*^*LSL-EYFP*^;FIC; *Bcl10*^*+/fl*^*;Rosa26*^*LSL-EYFP*^;FIC and *Bcl10*^*fl/fl*^*;Rosa26*^*LSL-EYFP*^;FIC were directly sorted into a 96-well PCR plate pre-filled with 10 µL of 1× TCL buffer (Qiagen) containing 1% (v v^−1^) β-mercaptoethanol (Sigma-Aldrich). Library preparation for bulk 3′-sequencing of poly(A)-RNA was performed as outlined by Parekh et al. ^50^. Briefly, for each sample, barcoded full-length cDNA was generated with a Maxima RT polymerase (Thermo Fisher) using oligo-dT primer containing barcodes, unique molecular identifiers (UMIs) and an adapter. The addition of a template switch oligo (TSO) resulted in the extension of 5′ ends of the cDNAs, and full-length cDNA was amplified with a primer binding to the TSO site and the adapter. cDNA was fragmented with the Nextera XT kit (Illumina), and only the 3′-end fragments were finally amplified using primers with Illumina P5 and P7 overhangs. In comparison to Parekh et al.^50^, the P5 and P7 sites were exchanged to allow sequencing of the cDNA in read1 and barcodes and UMIs in read2 to achieve a better cluster recognition. The library was sequenced on the NextSeq 500 platform (Illumina) with 75 cycles for the cDNA and 16 cycles for the barcodes and UMIs. Raw sequencing data were processed with DropSeq-tools version 1.12 using gene annotations from the Ensembl GRCm38.87 database to generate sample- and gene-wise UMI tables^51^. Downstream analysis was conducted with R v3.4.4^52^ and DESeq2 v1.18.1^53^. Technical replicates having <100,000 UMIs in total were excluded prior to differential expression analysis, and the remaining replicates were collapsed. Genes having <10 reads in total across all conditions were excluded. Prior differential expression analysis, dispersion of the data was estimated with a parametric fit including the genotype of the mice as explanatory variable in the model. Genes regulated between any of the three genotypes were determined with a likelihood ratio test. Genes with a false discovery rate (FDR) ≤10% were considered statistically significant. Hierachical clustering of regulated genes was conducted with the ward agglomeration method.

### Quantification and statistical analysis

The respective statistical test is indicated in each figure legend. In brief, we employed a Student’s *t* -test when only two groups were compared within a certain condition, while a one-way ANOVA combined with Tukey’s multiple comparisons test was used when more than two groups were compared. For testing a statistically significant difference between survival curves, we employed a log-rank (Mantel–Cox) test, whereas for comparison of tumor size the non-parametric Mann–Whitney was used. The GraphPad Prism 7.0c software was employed for testing the significance level of *α* = 0.05. For the calculation of median fluorescence intensity, FlowJo version 9.7.7 was employed. In order to find deregulated genes following RNA sequencing, we used a likelihood ratio test and considered genes with an FDR ≤10% as statistically significant.

### Reporting summary

Further information on research design is available in the Nature Research Reporting Summary linked to this article.

## Supplementary information

Supplementary Information

Reporting Summary

Source Data

## Data Availability

The RNA sequencing data that support the findings of this study have been deposited in the European Nucleotide Archive (ENA) under the accession code PRJEB32185. Source data underlying Figs. 1–7 and Supplementary Figs. 1, 3, and 5–9 are provided as a Source Data file with the paper. All other data are available from the authors upon reasonable requests.
