# Supplementary material for: Bcl10-controlled Malt1 paracaspase activity is key for the immune suppressive function of regulatory T cells
Source: Nat Commun. 2019 May 28;10:2352. doi: 10.1038/s41467-019-10203-2 (PMC6538646; doi:10.1038/s41467-019-10203-2)
Supplement: Supplementary file 1 — Supplementary Information [file 41467_2019_10203_MOESM1_ESM.pdf]

Bcl10-controlled Malt1 paracaspase activity is key for the immune suppressive function of regulatory T cells

Rosenbaum et al.

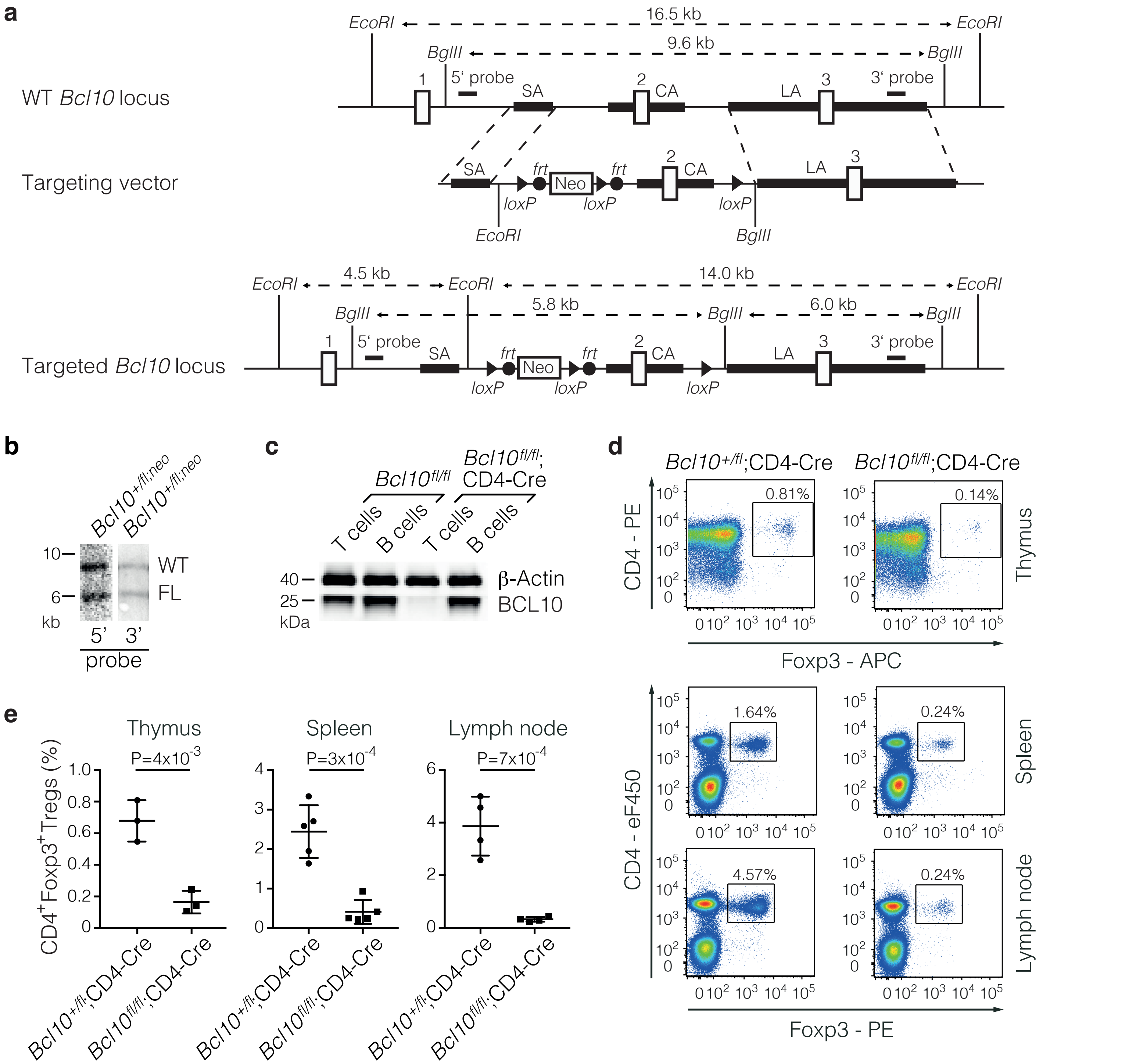

Supplementary Figure 1. Conditional gene targeting of *Bcl10*

(a) Structure of the targeted *Bcl10* locus on mouse chromosome 3. Thick black lines depict the homology regions, and rectangles represent the respective exons; black arrowheads indicate the *loxP* sites flanking exon #2, and filled dots mark the *frt* sites flanking the neomycin cassette. The restriction enzyme sites and 5’ and 3’ probes for Southern blot analysis are also depicted. (SA) short arm; (CA) conditional arm; (LA) long arm.

(b) Southern blot analysis of homologously recombined *Bcl10*$^{+/fl;neo}$ ES cells. DNA was digested with the restriction endonuclease *BglII* and either hybridized with a 5’ probe (left) to detect a 9.6 kb wild-type (WT) DNA fragment and a 5.8 kb DNA fragment of the floxed (FL) allele or with a 3’ probe resulting in a 9.6 kb WT DNA fragment and a 6 kb DNA fragment in the case of the targeted FL allele (right).

(c) Immunoblot of sorted splenic CD4$^+$ and CD8$^+$ T cells as well as splenic B220$^+$ B cells of either *Bcl10*$^{fl/fl}$ or *Bcl10*$^{fl/fl}$;CD4-Cre mice to detect the protein expression of BCL10. β-actin was used as a loading control.

(d) FACS analysis to detect the percentage of viable CD4$^+$Foxp3$^+$ Tregs in the thymus (upper panel), spleens (middle panel), and lymph nodes (lower panel) of adult *Bcl10*$^{fl/fl}$;CD4-Cre and *Bcl10*$^{+/fl}$;CD4-Cre control animals. The numbers indicate the percentage of cells within the gated quadrant and are representative of ≥3 mice per genotype.

(e) Freqencies of viable CD4$^+$Foxp3$^+$ Tregs in the thymus (left panel), spleens (middle panel), and lymph nodes (right panel) of adult *Bcl10*$^{fl/fl}$;CD4-Cre and *Bcl10*$^{+/fl}$;CD4-Cre control animals. The bars represent the mean +/-SD. Statistical significance between the means of the indicated genotypes was assessed by a two-tailed unpaired Student‘s *t*-test. Data of spleen and lymph nodes are cumulative from 3 independent experiments. Source data are provided as a Source Data file.

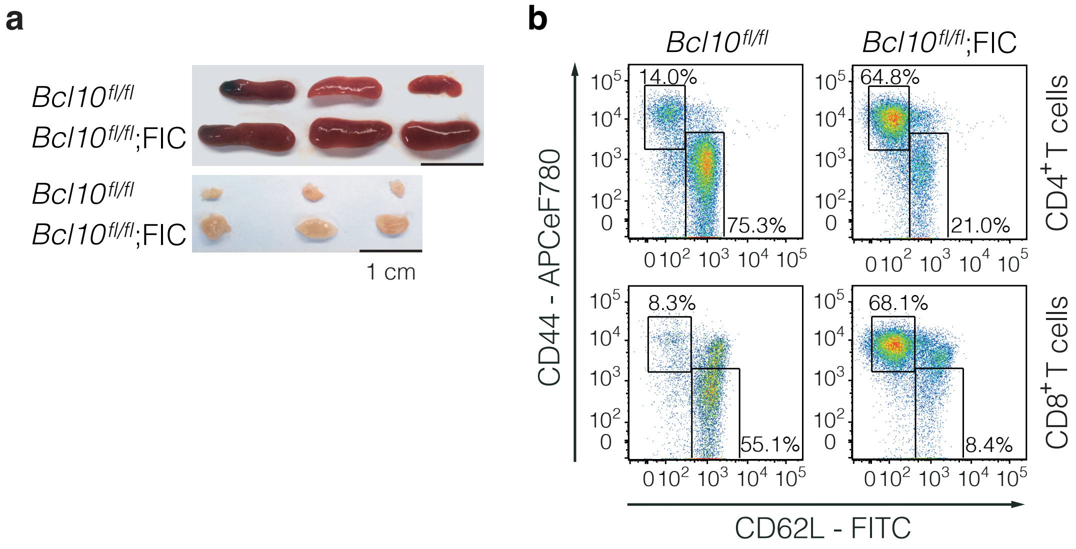

Supplementary Figure 2. Phenotype of *Bcl10*$^{fl/fl}$;FIC mice

(a) Size of spleens (upper panel) and lymph nodes (lower panel) of male *Bcl10*$^{fl/fl}$ control and *Bcl10*$^{fl/fl}$;FIC mice on day 16 post-partum. Scale bars represent 1 cm. Data are representative of 5 animals each.

(b) FACS profile of splenic CD4^{+}Foxp3^{-} T cells (upper panel) and CD8^{+} T cells to detect either CD44^{lo}CD62L^{hi} naïve or CD44^{hi}CD62L^{lo} effector T cells in 16-day-old male *Bcl10*$^{fl/fl}$ and *Bcl10*$^{fl/fl}$;FIC mice. The numbers indicate the percentage of cells within the quadrant and are representative of ≥7 16-day-old animals per genotype from 7 (upper panel) and 3 (lower panel) independent experiments.

Supplementary Figure 2

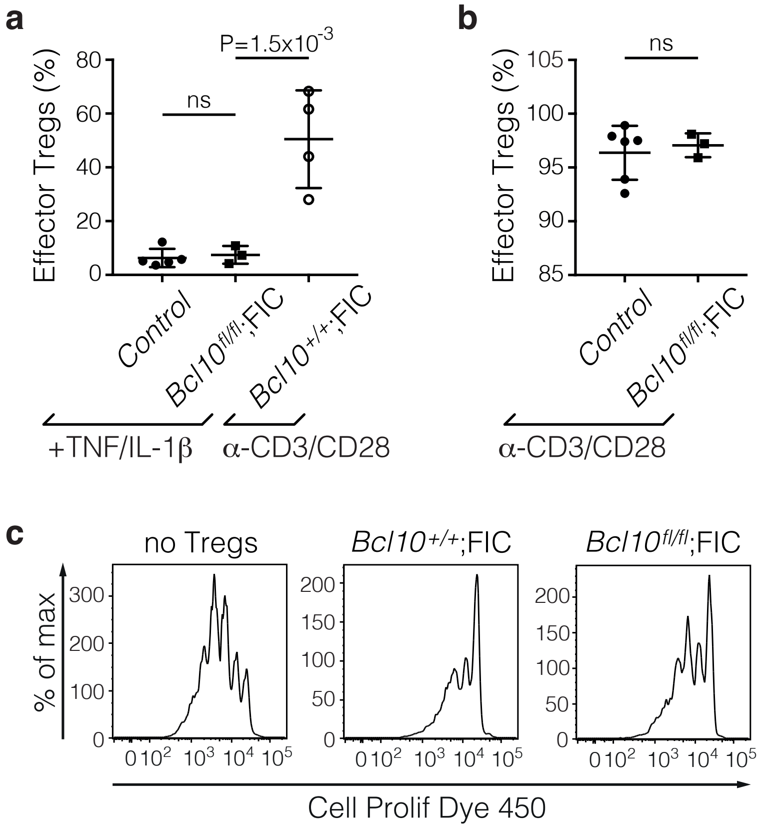

Supplementary Figure 3. BCL10-controlled Treg-suppressive phenotype

(a) Quantified analysis of the differentiation of sorted splenic CD4^{+}EYFP^{+}CD44^{lo}CD62L^{hi} naïve rTregs of diseased male *Bcl10*$^{fl/fl}$;*Rosa26*$^{LSL-EYFP}$;FIC and *Bcl10*$^{+/+}$;*Rosa26*$^{LSL-EYFP}$;FIC or *Bcl10*$^{+/fl}$;*Rosa26*$^{LSL-EYFP}$;FIC (control) mice into CD4^{+}EYFP^{+}CD44^{hi}CD62L^{lo} effector Tregs in the presence of TNF (20 ng mL^{-1}) and IL-1β (20 ng mL^{-1}); a 3 day anti-CD3/CD28 Dynabeads stimulation served as a differentiation control. Data are cumulative from 3 independent experiments and statistical significance between the conditions was assessed by ordinary one-way ANOVA combined with Tukey's multiple comparisons test.

(b) Quantified analysis of viable CD4^{+}EYFP^{+}CD44^{hi}CD62L^{-} effector Tregs following a 3 day anti-CD3/CD28 Dynabeads stimulation of sorted CD4^{+}EYFP^{+}CD44^{hi}CD62L^{-} effector Tregs of diseased male *Bcl10*$^{fl/fl}$;*Rosa26*$^{LSL-EYFP}$;FIC and *Bcl10*$^{+/+}$ or *Bcl10*$^{+/fl}$;*Rosa26*$^{LSL-EYFP}$;FIC (control) mice. Data are cumulative from 3 independent experiments. Statistical significance between the genotypes was assessed by a two-tailed unpaired Student's *t*-test.

(c) *In vitro* Treg suppressor assay assessing the suppressor activity of eTregs of *Bcl10*$^{+/+}$ and *Bcl10*$^{fl/fl}$;*Rosa26*$^{LSL-EYFP}$;FIC mice. Sorted splenic CD4^{+}CD25^{-}CD45RB^{hi} naïve conventional T cells were labeled with Cell Proliferation Dye eFluor 450 and cultivated for 3 days without any Tregs (left panel), with sorted splenic CD4^{+}EYFP^{+}CD44^{hi}CD62L^{lo} eTregs of male *Bcl10*$^{+/+}$;*Rosa26*$^{LSL-EYFP}$;FIC mice (middle panel) or *Bcl10*$^{fl/fl}$;*Rosa26*$^{LSL-EYFP}$;FIC mice (right panel) in the presence of irradiated splenocytes and soluble anti-CD3. The FACS plots are representative of 2 independent experiments and show the proliferation profile of viable cells cultivated at a 2:1 ratio with Tregs.

Bars in (a) and (b) indicate the mean +/- SD. Source data are provided as a Source Data file.

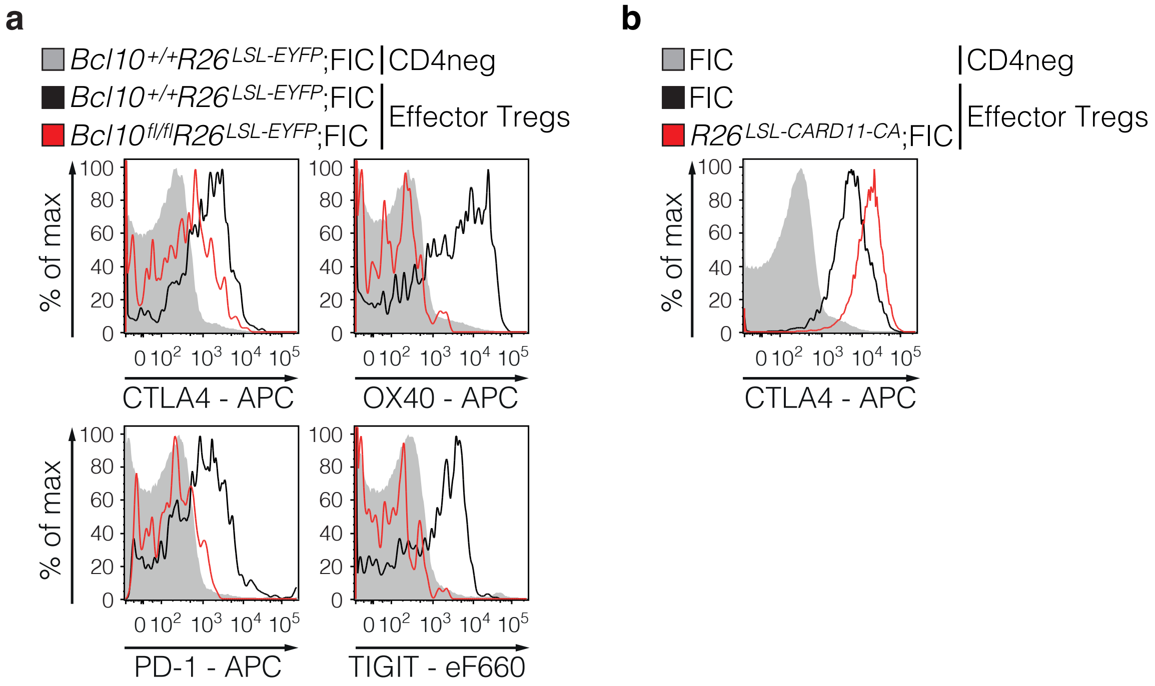

Supplementary Figure 4. BCL10-controlled Treg-suppressive phenotype

(a) Representative FACS analysis to detect the expression of CTLA4, OX40, PD-1, and TIGIT on viable CD4$^+$Foxp3$^+$EYFP$^+$CD44^{hi}CD62L^{lo}-gated effector Tregs in the spleens of either *Bcl10$^{fl/fl}$Rosa26$^{LSL-EYFP}$*;FIC mice (red line, n=4) or *Bcl10$^{+/+}$Rosa26$^{LSL-EYFP}$*;FIC control mice (black line, n=4). CD4$^-$ cells of *Bcl10$^{+/+}$Rosa26$^{LSL-EYFP}$*;FIC mice (gray histogram) were gated as a negative expression control.

(b) Representative FACS plots to detect the expression of CTLA4 in viable CD4$^+$Foxp3$^+$CD44^{hi}CD62L^{lo}-gated effector Tregs in the spleens of adult FIC control mice (black line, n=4) or *Rosa26$^{LSL-CARD11-CA}$*;FIC mice (red line, n=4). In the case of the latter mice, the cells were also gated for GFP. CD4$^-$ cells of adult FIC control mice (gray histogram) were gated as a negative expression control.

Data are representative of 2 independent experiments with 2 mice per genotype.

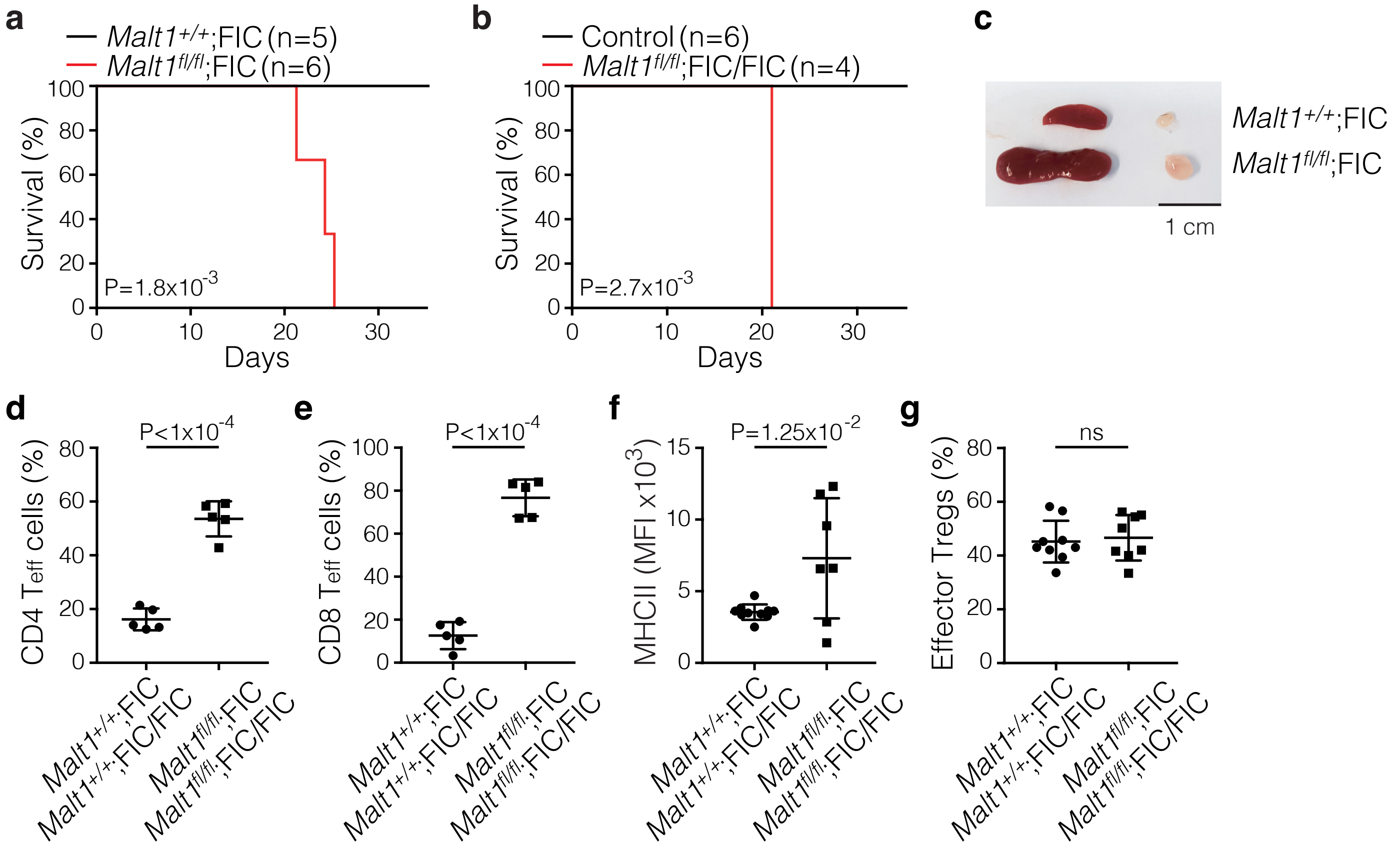

Supplementary Figure 5. ***Malt1*** **in mature peripheral Tregs prevents autoimmune inflammation**

(a and b) Survival curves of (a) male *Malt1*$^{+/+}$;FIC (black line, n=5) versus *Malt1*$^{fl/fl}$;FIC mice (red line, n=6) and (b) female *Malt1*$^{+/+}$;FIC/FIC and *Malt1*$^{+/fl}$;FIC/FIC control (black line, n=6) versus *Malt1*$^{fl/fl}$;FIC/FIC (red line, n=4) mice. Survival equals the day the animal had to be sacrificed to avoid severe burden. Statistical significance between the survival curves with the corresponding p-value was calculated by a log-rank (Mantel-Cox) test.

(c) Size of spleen (left) and lymph node (right) in male *Malt1*$^{fl/fl}$;FIC mice and *Malt1*$^{+/+}$;FIC control mice, aged 21 days. Data are representative of 2 animals each. Scale bars represent 1 cm.

(d and e) Frequencies of (d) CD4^{+}Foxp3^{-} and (e) CD8^{+} CD44^{hi}CD62L^{lo}-gated effector T (Teff) cells in the spleens of male *Malt1*$^{+/+}$;FIC (n=3) and female *Malt1*$^{+/+}$;FIC/FIC (n=2) control mice versus diseased male *Malt1*$^{fl/fl}$;FIC (n=2) and female *Malt1*$^{fl/fl}$;FIC/FIC (n=3) mice. Data are cumulative from 2 independent experiments.

(f) Median fluorescence intensity (MFI) of cell surface MHCII on CD19^{+}-gated splenic B cells of male *Malt1*$^{+/+}$;FIC (n=6) and female *Malt1*$^{+/+}$;FIC/FIC (n=4) control mice versus diseased male *Malt1*$^{fl/fl}$;FIC (n=3) and female *Malt1*$^{fl/fl}$;FIC/FIC (n=4) mice.

(g) Quantified frequency of CD44^{hi}CD62L^{lo} effector Tregs in the viable CD4^{+}Foxp3^{+} Treg population in the spleens of male *Malt1*$^{+/+}$;FIC (n=5) and female *Malt1*$^{+/+}$;FIC/FIC (n=4) control mice versus diseased male *Malt1*$^{fl/fl}$;FIC (n=3) and female *Malt1*$^{fl/fl}$;FIC/FIC (n=5) mice.

Data in (f) and (g) are cumulative from 5 independent experiments. Bars in (d)-(g) indicate the mean +/- SD. Statistical significances between the genotypes in (d)-(g) were assessed by a two-tailed unpaired Student's *t*-test. Significance values are depicted in the graph; (ns) not significant.

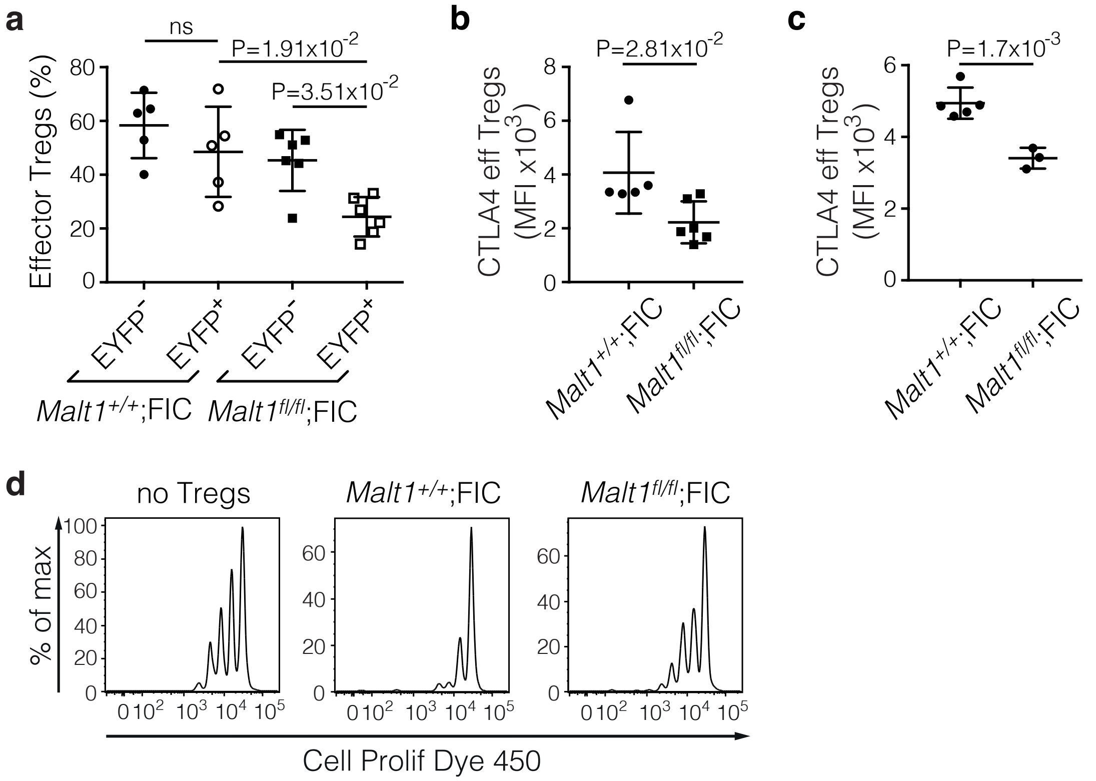

Supplementary Figure 6. MALT1 regulates the homeostatic rTreg to eTreg conversion and mediates Treg suppression

(a) Frequency of EYFP⁻ and EYFP⁺-gated CD44^{hi}CD62L^{lo} effector Tregs in the viable CD4⁺Foxp3⁺ Treg population in the spleens of female *Malt1*$^{+/+}$;*Rosa26*$^{LSL-EYFP}$;FIC and *Malt1*$^{fl/fl}$;*Rosa26*$^{LSL-EYFP}$;FIC mice. Statistical significance between the indicated genotypes was assessed by ordinary one-way ANOVA combined with Tukey's multiple comparisons test. Data are cumulative from 3 independent experiments.

(b and c) Quantified analysis of the median fluorescence intensity (MFI) of CTLA4 on viable (b) CD4⁺Foxp3⁺CD44^{hi}CD62L^{lo}-gated EYFP⁺ effector (eff) Tregs in the spleens of adult *Malt1*$^{+/+}$;*Rosa26*$^{LSL-EYFP}$;FIC and *Malt1*$^{fl/fl}$;*Rosa26*$^{LSL-EYFP}$;FIC female mice, and (c) CD4⁺Foxp3⁺CD44^{hi}CD62L^{lo}-gated eff Tregs in the spleens of diseased male *Malt1*$^{fl/fl}$;FIC and *Malt1*$^{+/+}$;FIC control mice. Data are cumulative from (b) 3 and (c) 2 independent experiments.

(d) *In vitro* Treg suppressor assay assessing the suppressor activity of Tregs of *Malt1*$^{+/+}$ and *Malt1*$^{fl/fl}$;*Rosa26*$^{LSL-EYFP}$;FIC mice. Sorted splenic CD4⁺CD25⁻CD45RB^{hi} naïve conventional T cells were labeled with Cell Proliferation Dye eFluor 450 and cultivated for 3 days without any Tregs (left panel), with sorted splenic CD4⁺EYFP⁺ Tregs of *Malt1*$^{+/+}$;*Rosa26*$^{LSL-EYFP}$;FIC (middle panel) or *Malt1*$^{fl/fl}$;*Rosa26*$^{LSL-EYFP}$;FIC mice (right panel) in the presence of irradiated splenocytes and soluble anti-CD3. The FACS plots are representative of 2 independent experiments each comprising n≥2 mice per genotype and show the proliferation profile of viable cells cultivated at a 2:1 ratio with Tregs.

Bars in (a)-(c) indicate the mean +/- SD. Statistical significances between the genotypes in (b) and (c) were assessed by a two-tailed unpaired Student's *t*-test. Significance values are depicted in the graph; (ns) not significant. Source data are provided as a Source Data file.

Supplementary Figure 6

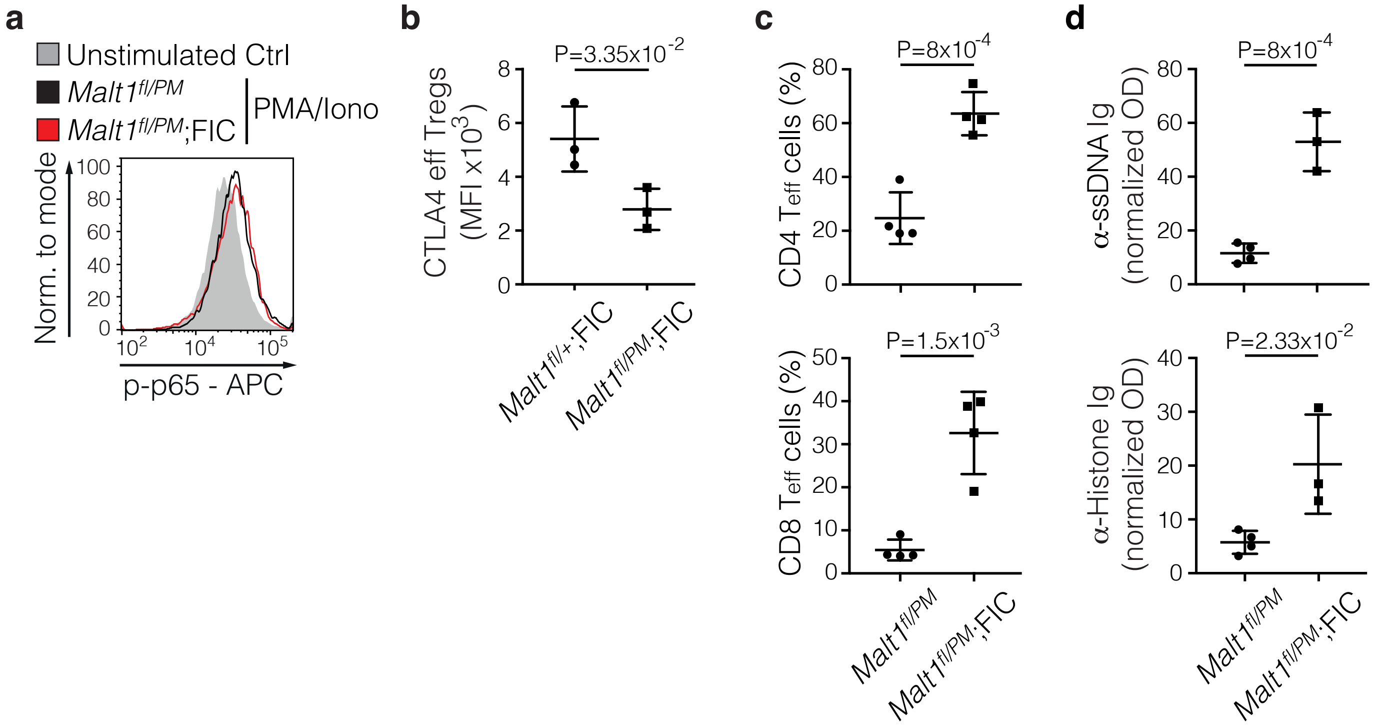

Supplementary Figure 7. MALT1 paracaspase function in Tregs

(a) PhosFlow experiment to detect the median fluorescence intensity (MFI) of phospho (p)-NFκB p65 at serine 536 in either unstimulated (gray histogram) or 30 min PMA/ionomycin-stimulated viable $CD4^+Foxp3^+$-gated Tregs of diseased *Malt1*$^{fl/PM}$;FIC mice (red line) and respective *Malt1*$^{fl/PM}$ littermate control mice (black line). Data are representative of 2 independent experiments comprising 2 mice per genotype.

(b) Quantified analysis of CTLA4 MFI on $CD4^+Foxp3^+CD44^{hi}CD62L^{lo}$-gated $EYFP^+$ effector (eff) Tregs in the spleens of adult *Malt1*$^{fl/+}$;*Rosa26*$^{LSL\text{-}EYFP}$;FIC and *Malt1*$^{fl/PM}$;*Rosa26*$^{LSL\text{-}EYFP}$;FIC female mice.

(c) Quantified analysis of the percentage of $CD44^{hi}CD62L^{lo}$-gated $CD4^+Foxp3^-$ (upper panel) and CD8 (lower panel) T effector cells (Teff) in the spleens of *Malt1*$^{fl/PM}$ and diseased *Malt1*$^{fl/PM}$;FIC mice on the day of sacrifice according to the survival curve shown in Figure 6k.

(d) Quantification of anti-ssDNA (upper graph) and anti-histone immunoglobulins (lower graph) in the sera of diseased *Malt1*$^{fl/PM}$;FIC and *Malt1*$^{fl/PM}$ control mice on the day of sacrifice according to the survival curve.

Data in (b) and (c) are cumulative from 2 independent experiments. Bars in (b)-(d) indicate the mean +/- SD and statistical significances were assessed by a two-tailed unpaired Student's *t*-test. Source data are provided as a Source Data file.

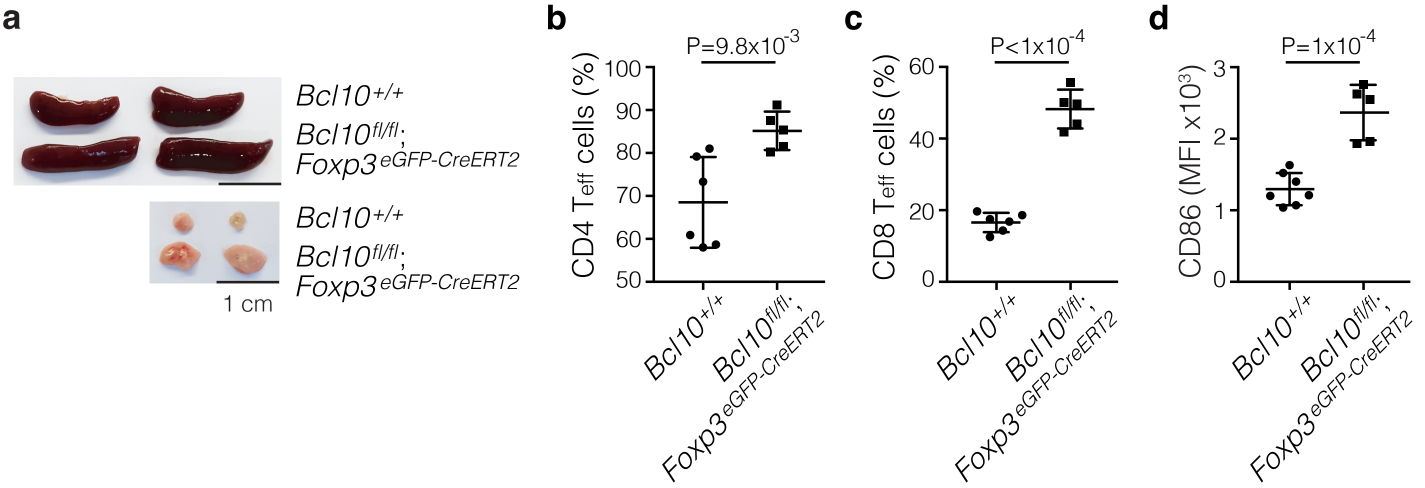

Supplementary Figure 8. The induced deletion of *Bcl10* in Tregs results in systemic immune activation

(a) Size of spleens (upper panel) and lymph nodes (lower panel) of adult *Bcl10*$^{+/+}$;*Foxp3*$^{eGFP\text{-}CreERT2}$ and *Bcl10*$^{fl/fl}$;*Foxp3*$^{eGFP\text{-}CreERT2}$ mice on day 28 after tamoxifen treatment. Data are representative of ≥5 animals each; scale bars represent 1 cm.

(b and c) Frequency of splenic (b) $CD4^+Foxp3^-$ and (c) $CD8^+$ $CD44^{hi}CD62L^{lo}$ effector T cells (Teff) in adult *Bcl10*$^{+/+}$;*Foxp3*$^{eGFP\text{-}CreERT2}$ and *Bcl10*$^{fl/fl}$;*Foxp3*$^{eGFP\text{-}CreERT2}$ mice on day 28 after tamoxifen treatment as measured by FACS.

(d) Median fluorescence intensity (MFI) of cell surface CD86 on $CD19^+B220^+$-gated splenic B cells of adult *Bcl10*$^{+/+}$;*Foxp3*$^{eGFP\text{-}CreERT2}$ and *Bcl10*$^{fl/fl}$;*Foxp3*$^{eGFP\text{-}CreERT2}$ mice on day 28 after tamoxifen treatment.

Bars in (b)-(d) indicate the mean +/- SD and statistical significances were assessed by a two-tailed unpaired Student's *t*-test. Source data are provided as a Source Data file.

Supplementary Figure 8

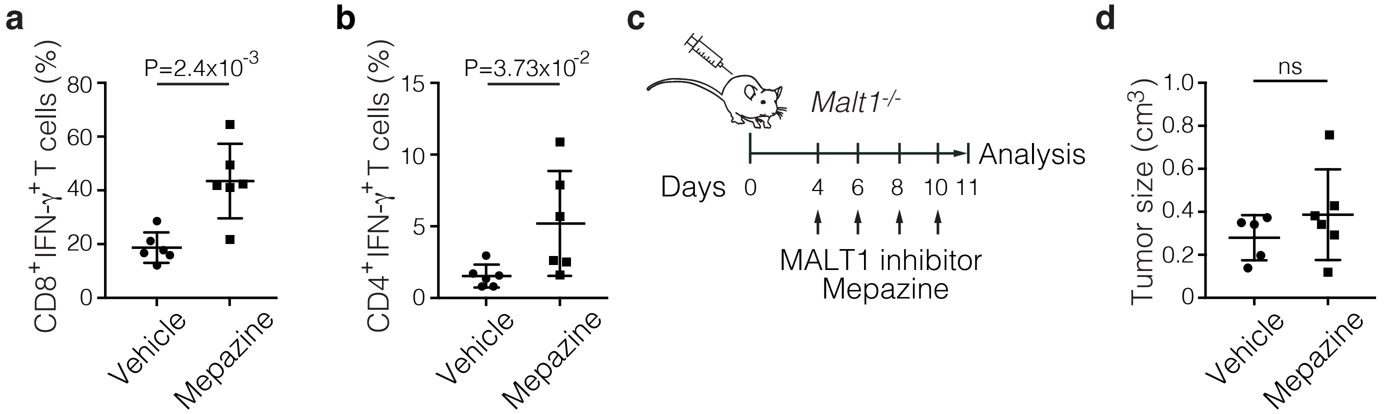

Supplementary Figure 9. Inhibition of MALT1 paracaspase function enhances anti-tumor immunity

(a and b) Qantification of the ratio of tumor-infiltrating (b) CD8⁺IFN-γ⁺ cells and (a) CD4⁺Foxp3⁻IFN-γ⁺ cells to the total frequency of T cells (CD4⁺ plus CD8⁺) after re-stimulation of enriched tumor-infiltrating lymphocytes with PMA/ionomycin. Statistical significance between means was assessed by a two-tailed unpaired Student's *t*-test.

(c) Schematic representation of the B16-OVA tumor model in *Malt1*$^{-/-}$ mice combined with a pharmacological inhibition of the MALT1 protease activity: B16-OVA cells were subcutaneously injected into the flanks of *Malt1*$^{-/-}$ mice, followed by treatment with either the MALT1 inhibitor mepazine or vehicle from day 4 onwards every other day.

(d) Tumor size on the final analysis day 11 following treatment of either vehicle (PBS/5% DMSO) or mepazine (16 mg kg^{-1} body weight) from day 4 onwards every other day. A statistical significance between groups was assessed by a two-tailed Mann-Whitney U test.

Bars in (a), (b), and (d) represent the mean +/-SD. Source data are provided as a Source Data file.

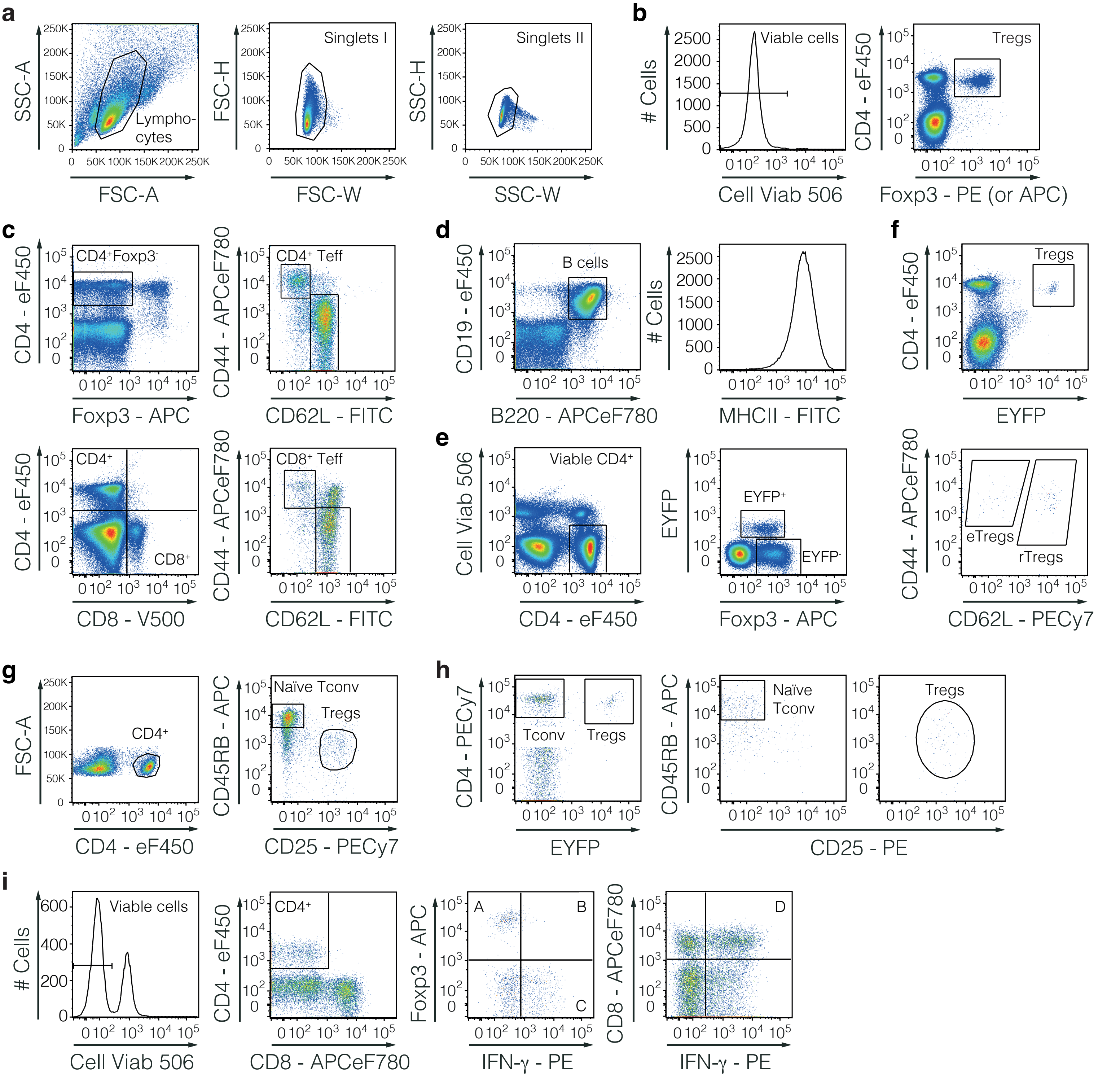

Supplementary Figure 10. Gating strategies

(a) Basic gating strategy for single lymphocytes in all FACS plots or sorts.

(b) Gating strategy for viable $CD4^+Foxp3^+$ Tregs in Fig. 1a, 2f, 2g, and Supplementary Fig. 1d and e. Further gating into effector Tregs was performed as indicated in Fig. 3e for Fig. 3f, 5e, 6d, 6f, 6g and Supplementary Fig. 5g and 6c.

(c) Strategy to gate $CD4^+$ and $CD8^+$ effector T cells (Teff) in Fig. 1e, 4b, 6i and in Supplementary Fig. 5d, 5e, 7c, 8b and c.

(d) Exemplified gating strategy for splenic B cells to detect the mean fluoroscence intensities of MHCII and CD86 in Fig. 1f, 4b and Supplementary Fig. 5f and 8d.

(e) Gating strategy to distinguish $EYFP^-$/$EYFP^+$ Tregs in Fig. 2b and c and transgenic GFP^+ Tregs in Fig. 2h and i. Further effector Treg gating as exemplified in Fig. 2b was performed in Fig. 5b, 5d, 6e and Supplementary Fig. 4a, 4b, 6a, 6b and 7b.

(f) Gating strategy to sort $CD4^+EYFP^+$ Tregs in Fig. 5h and i, and naïve $CD4^+EYFP^+CD62L^{hi}$ rTregs or $CD4^+EYFP^+CD44^{hi}CD62L^{lo}$ eTregs in Fig. 2d, 2e, 3d, 5f, 5g and Supplementary Fig. 3a and b. For the acquisition of differentiated cells, Cell viability Dye 506 gating was included and preceded gating for Tregs. Same was done to sort eTregs for RNA sequencing in Fig. 5a.

(g) Gating strategy to sort $CD4^+CD25^-CD45RB^{hi}$ naïve conventional T cells (Tconv) and $CD4^+CD25^+CD45RB^{lo}$ Tregs in Fig. 3g.

(h) Gating strategy to sort $CD4^+CD25^-CD45RB^{hi}$ naïve Tconv and $CD4^+EYFP^+$ Tregs for the immunoblot depicted in Fig. 6a and the *in vitro* Treg suppressor assays in Fig. 6l and Supplementary Fig. 6d.

(i) Gating strategy to determine the percentages of $CD4^+Foxp3^+$ Tregs (A+B) (Fig. 7g) and $CD4^+Foxp3^-IFN\text{-}\gamma^+$ cells (C) (Fig. 7e and Supplementary Fig. S9b). Cell gating for $CD8^+IFN\text{-}\gamma^+$ cells (D) (Fig. 7d and Supplementary Fig. 9a) from the viable cell population.

Supplementary Figure 10

**Gene locus/ allele**	**Primer name**	**Primer sequence**
Bcl10floxed	cB10 fwd	5'-CTA TCT GTA CCA ACA ACT GAA GAG-3'
	cB10 rev	5'-ATA CAG GAT TCT TAC ACA CTC GT-3'
CD4-Cre	Cre fwd	5'-ACC AGC CAG CTA TCA ACT CG-3'
	Cre rev	5'-TTA CAT TGG TCC AGC CAC C-3'
Foxp3 wt	Wt-FIC-rev	5'-TTC GCA AGA AGA GGA GCC AAC-3'
	Wt-FIC SH_38	5'-GGC CAA GGC AGA TAT GTA TAG C-3'
Foxp3-IRES-Cre (FIC)	FIC SH_37	5'-CCC CAA CAA GTG CTC CAA TCC-3'
	FIC_fwd	5'-CTG CTT CTT TCA CGA CAT TCA AG-3'
Foxp3eGFP-CreERT2	14125 = Foxp3creERT2 wt fwd	5'-CCT AGC CCC TAG TTC CAA CC-3'
	14126 = Foxp3creERT2 wt rev	5'-AAG GTT CCA GTG CTG TTG CT-3'
	14127 = Foxp3creERT2 mut fwd	5'-TTA CGG CGC TAA GGA TGA CT-3'
	15302 = Foxp3creERT2 mut rev	5'-TCA CTG AAG GGT CTG GTA GGA-3'
Malt1 wt/ko	Malt_neo	5'-GGG TGG GAT TAG ATA AAT GCC TGC TC-3'
	Malt_com	5'-CTG CTG CTG ACA TGC TAC AAT ATG CTG-3'
	Malt_wt	5'-ACT TTC ATC TTG CCA GCA CTC TTT CTT A-3'
PM	PM fwd	5'-CTG GTG GCA CAC ACT TTT AG-3'
	PM rev	5'-CCA ACA TAC ATA CGA ATG GAC-3'
Malt1floxed	cM1_wtfloxdel_fwd	5'-CTA GTC AGT CAC CAG CTC AG-3'
	cM1_wtflox_rev	5'-CAG TTC TCA ATG CCA ACG CAC-3'
	cM1_del_rev	5'-CTG GCT AAC CAA TCC TCA AAA C-3'
Rosa26-LSL-EYFP	R26 eYFP SH 204	5'-AAG ACC GCG AAG AGT TTG TC-3'
	R26 eYFP SH 205	5'-AAA GTC GCT CTG AGT TGT TAT-3'
	R26 eYFP SH 206	5'-GGA GCG GGA GAA ATG GAT ATG-'3
Rosa26-LSL-CARD11-CA	IRES	5'-ATA CGC TTG AGG AGA GCC ATT TG-3'
	Carma mm	5'-AAG GAC AAG ATC GGC GAG GAG-3'
	long geno 30	5'-ACT CGG GTC AGC ATG TCT TTA ATC-3'
	short geno 31	5'-GTG ATC TGC AAC TCC AGT CTT TCT A-3'
Rosa26-LSL-IKK2-CA	790 rosa forw	5'-AAA GTC GCT CTG AGT TGT TAT C-3'
	791 rosa rev	5'-GAT ATG AAG TAC TGG GCT CTT-3'
	792 neo rev	5'-GCA TCG CCT TCT ATC GCC T-3'

Supplementary Table 1. Primers used for genotyping

Supplementary Table 1
