# Supplementary material for: Bcl10-controlled Malt1 paracaspase activity is key for the immune suppressive function of regulatory T cells
Source: Nat Commun. 2019 May 28;10:2352. doi: 10.1038/s41467-019-10203-2 (PMC6538646; doi:10.1038/s41467-019-10203-2)
Supplement: Supplementary file 2 — Reporting Summary [file 41467_2019_10203_MOESM2_ESM.pdf]

nature research

Corresponding author(s): Jürgen Ruland

Reporting Summary

Nature Research wishes to improve the reproducibility of the work that we publish. This form provides structure for consistency and transparency in reporting. For further information on Nature Research policies, see Authors & Referees and the Editorial Policy Checklist.

Statistical parameters

When statistical analyses are reported, confirm that the following items are present in the relevant location (e.g. figure legend, table legend, main text, or Methods section).

n/a	Confirmed	
☐	☒	The exact sample size (n) for each experimental group/condition, given as a discrete number and unit of measurement
☐	☒	An indication of whether measurements were taken from distinct samples or whether the same sample was measured repeatedly
☐	☒	The statistical test(s) used AND whether they are one- or two-sided<br>*Only common tests should be described solely by name; describe more complex techniques in the Methods section.*
☐	☒	A description of all covariates tested
☐	☒	A description of any assumptions or corrections, such as tests of normality and adjustment for multiple comparisons
☐	☒	A full description of the statistics including central tendency (e.g. means) or other basic estimates (e.g. regression coefficient) AND variation (e.g. standard deviation) or associated estimates of uncertainty (e.g. confidence intervals)
☐	☒	For null hypothesis testing, the test statistic (e.g. F, t, r) with confidence intervals, effect sizes, degrees of freedom and P value noted<br>*Give P values as exact values whenever suitable.*
☒	☐	For Bayesian analysis, information on the choice of priors and Markov chain Monte Carlo settings
☐	☒	For hierarchical and complex designs, identification of the appropriate level for tests and full reporting of outcomes
☐	☒	Estimates of effect sizes (e.g. Cohen's d, Pearson's r), indicating how they were calculated
☐	☒	Clearly defined error bars<br>*State explicitly what error bars represent (e.g. SD, SE, CI)*

Our web collection on statistics for biologists may be useful.

Software and code

Policy information about availability of computer code

Data collection	Flow Cytometry: FACSDiva (BD Biosciences) for FACS CantoII und FACS Aria III, Ideas 6.2.183.0 (Merck) for Amnis Image Stream<br>WES (Protein Simple) Western blot: Compass for SW Vers. 3.1.7<br>Histology: Olympus BX53 with CellSens Dimension (Olympus) or AxioCam with AxioVision (Carl Zeiss)<br>RNAseq: NextSeq 500 (Illumina)
Data analysis	RNAseq analysis: DropSeq-tools version1.12, R v3.4.4, DESeq2v1.18.1<br>Flow cytometry: FlowJo 9.7.7; FlowJo 10.1r7, Ideas 6.2.183.0 (Merck) and FCSExpress6Plus for Amnis Image Stream<br>Data visualization: GraphPad Prism 5 and 7

For manuscripts utilizing custom algorithms or software that are central to the research but not yet described in published literature, software must be made available to editors/reviewers upon request. We strongly encourage code deposition in a community repository (e.g. GitHub). See the Nature Research guidelines for submitting code & software for further information.

Data

Policy information about availability of data

All manuscripts must include a data availability statement. This statement should provide the following information, where applicable:

- Accession codes, unique identifiers, or web links for publicly available datasets
- A list of figures that have associated raw data
- A description of any restrictions on data availability

The RNA sequencing data that support the findings of this study have been deposited in the European Nucleotide Archive (ENA) under the accession code PRJEB32185 [https://www.ebi.ac.uk/ena/data/search?query=PRJEB32185].

Field-specific reporting

Please select the best fit for your research. If you are not sure, read the appropriate sections before making your selection.

☒ Life sciences ☐ Behavioural & social sciences ☐ Ecological, evolutionary & environmental sciences

For a reference copy of the document with all sections, see nature.com/authors/policies/ReportingSummary-flat.pdf

Life sciences study design

All studies must disclose on these points even when the disclosure is negative.

Sample size	No statistical analysis methods were used to predetermine sample size estimates. For in vivo tumor experiments, a minimal sample size of n=5 mice were used to ensure a sufficient number for statistical analysis.
Data exclusions	No data were excluded from the analyses.
Replication	All experimental findings were reliably reproduced as indicated in the figure legends.
Randomization	No randomization was performed except of the B16-OVA tumor experiment, in which on day 11, tumors were measured with a caliber and homogenously allocated to the vehicle and Mepazine group to exclude a bias.
Blinding	Blinding was not performed.

Reporting for specific materials, systems and methods

Materials & experimental systems

n/a	Involved in the study
☐	☒ Unique biological materials
☐	☒ Antibodies
☐	☒ Eukaryotic cell lines
☒	☐ Palaeontology
☐	☒ Animals and other organisms
☒	☐ Human research participants

Methods

n/a	Involved in the study
☒	☐ ChIP-seq
☐	☒ Flow cytometry
☒	☐ MRI-based neuroimaging

Unique biological materials

Policy information about availability of materials

Obtaining unique materials	All unique materials used are available from the authors upon reasonable request.

Antibodies

Antibodies used	Purified anti-CD16/32 clone 93 (eBioscience, 14-0161-86), anti-B220-APCeFluor780 clone RA3-6B2 (eBioscience, 47-0452-80), anti-CD4-eFluor450 clone GK1.5 (eBioscience, 48-0042-80), anti-CD4-PECy7 clone GK1.5 (eBioscience, 25-0041-82), anti-CD4-PE clone GK1.5 (eBioscience, 12-0042-82), anti-CD8 BD Horizon V500 clone 53-6.7 (BD Biosciences, 560776), anti-CD8-APCeFluor780 clone 53-6.7 (eBiosciene, 47-0081-82), anti-CD19-eFluor450 clone 1D3 (eBioscience, 48-0193-80), anti-CD25-

PECy7 clone PC61.5 (eBioscience, 25-0251-81), anti-CD44-APCeFluor780 clone IM7 (eBioscience, 47-0041-80), anti-CD44-PerCPCy5.5 clone IM7 (eBioscience, 45-0441-80), anti-CD45RB-APC clone C363.16A (eBioscience, 17-0455-81), anti-CD62L-FITC clone MEL.14 (eBioscience, 11-0621-82), anti-CD62L-PECy7 clone MEL.14 (eBioscience, 25-0621-81), anti-CD86-PECy7 clone GL1 (eBioscience, 15-0862-82), anti-CTLA4(CD152)-APC clone UC10-4B9 (eBioscience, 17-1522-82), anti-Foxp3-eFluor450 clone FJK.16s (eBioscience, 48-5773-80), anti-Foxp3-PE clone FJK.16s (eBioscience, 12-5773-80), anti-Foxp3-APC clone FJK.16s (eBioscience, 17-5773-80), anti-IFN-γ-PE clone XMG1.2 (eBioscience, 12-7311-81), anti-MHCII-FITC clone M5/ 114.15.2 (eBioscience, 11-5321-81), anti-OX40-APC (CD134) clone OX86 (eBioscience 17-341-80), anti-PD-1(CD279)-APC clone J43 (eBioscience, 17-9985-80), anti-TIGIT-eFluor660 clone GIGD7 (eBioscience, 50-9501-80), anti-phospho-NFKBp65 (Ser536) clone 93H1 Cell Signaling, #3033S), anti-rabbit IgG-APC (Invitrogen, A10931), anti-NFKB p65 (SantaCruz Biotechnology, sc-372), anti-cRel (Santa Cruz Biotechnology, sc-71), anti-rabbit IgG-FITC (BD Pharmingen, 554020), purified anti-CD3e clone 145-2C11 (eBioscience, 16-0031-85), anti-BCL10 clone C78F1 (CellSignaling, #4237), anti-β-Actin clone 8H10D10 (Cell Signaling, #3700), anti-Regnase-1 clone 604421 (R&D Systems, MAB7875) , anti-Roquin rat hybridoma clone 3F12 (Vogel et al., Immunity 2013), anti-p-Erk (Cell Signaling, clone E19 #9106), anti-GAPDH clone 6C5 (Calbiochem), anti-mouse IgG-HRP (Cell Signaling, #7076), anti-rat IgG-HRP (GE Healthcare, #NA935V), anti-rabbit IgG-HRP (Cell Signaling, #7074).

Validation

All antibodies were validated for the application and species used in this study by their manufacturers or by the mentioned research publication.

Eukaryotic cell lines

Policy information about cell lines

Cell line source(s)

B16F1 was purchased from ATCC (CRL-6323), whereas B16-OVA cells were obtained from the laboratory of C. Kurts (Institute of Experimental Immunology, Rheinische-Friedrichs-Wilhelms University of Bonn, Germany; original source: Brown, D. M., Fisher, T. L., Wei, C., Frelinger, J. G. & Lord, E. M. Tumours can act as adjuvants for humoral immunity. Immunology 102, 486-497 (2001).)

Authentication

B16F1 cell line was authenticated by ATCC.

Mycoplasma contamination

All cell lines were routinely tested for mycoplasma infection.

Commonly misidentified lines
(See ICLAC register)

No commonly misidentified cell line was used.

Animals and other organisms

Policy information about studies involving animals; ARRIVE guidelines recommended for reporting animal research

Laboratory animals

The sex and age of the used mice is indicated in the figure legends; if not stated, adult mice aged 6-12 weeks were used for all experiments; littermate controls were used whenever possible. CD4-Cre (Jackson 022071), Foxp3eGFP-Cre-ERT2 (Jackson 016961), Rosa26-LSL-EYFP (Jackson 006148) and Rosa26-LSL-IKK2-CA (Jackson 008242) were on the genetic background indicated by the Jackson laboratory. Bcl10floxed were generated as a mixed 129X1x129S1xC57BL/6J background and were crossed for at least four generations onto C57BL/6N background. Foxp3-IRES-Cre (FIC), Malt1floxed, Malt1-PM and Rosa26-LSL-CARD11-CA mice were on a C57BL/6N background. C57BL/6N mice were purchased from Charles River. All mice were kept under specific pathogen free condition.

Wild animals

The study did not involve wild animals.

Field-collected samples

The study did not involve samples collected from the field.

Flow Cytometry

Plots

Confirm that:

☒ The axis labels state the marker and fluorochrome used (e.g. CD4-FITC).

☒ The axis scales are clearly visible. Include numbers along axes only for bottom left plot of group (a 'group' is an analysis of identical markers).

☒ All plots are contour plots with outliers or pseudocolor plots.

☒ A numerical value for number of cells or percentage (with statistics) is provided.

Methodology

Sample preparation

Spleen, thymus and axillary and inguinal lymph nodes were harvested per mouse. Organs were meshed through a 70 µm cell strainer in 5 mL FACS buffer (PBS+2%FCS) and erythrocytes were lysed using G-DEXTMIIb RBC Lysis Buffer (Intron Biotechnologies). For enrichment of tumor-infiltrating lymphocytes (TILs), tumor samples were cut into small pieces and digested for 30 min at 37°C with 2 mg/mL Collagenase D (Roche) and 50 µg/mL DNase I (Roche) in RPMI 1640 (Thermo Fisher Scientific) supplemented with 10% (v/v) FCS (Capricorn Scientific), 1% (v/v) Penicillin-Streptomycine-Glutamine (Thermo Fisher Scientific), 1mM Sodium Pyruvate (Thermo Fisher Scientific), 10 mM HEPES (Thermo Fisher Scientific), 1X Gibco® MEM NE-AA (Thermo Fisher Scientific) and 56 µM ß-mercaptoethanol (Thermo Fisher Scientific). Following addition of a final concentration of 10 mM EDTA, cells were meshed and TILs were enriched at the interface using a 36%/80% Percoll (GE healthcare) gradient.

Cells were counted using a hemocytometer and 2 million cells per lymphoid organ and antibody-stain were plated into a 96-V-bottom plate. Following one washing steps with 1x PBS, cells were resuspended for 15 min in Fixable Viability Dye eFluor 450 or 506 (eBioscience) at 4°C in the dark. Cells were washed once with FACS buffer and resuspended in anti-CD16/32 antibodies for Fc receptor blocking. After 7 min incubation , cells then resuspended in antibody mixtures for surface staining.
For intracellular FACS, cells were fixed with 2% Formalin for 40 min, permeabilized by two washes with 1X Perm/Wash buffer (eBioscience) and stained overnight with the fluorochrome-coupled antibodies in 1X Perm/Wash buffer. For Phosflow, splenocytes were either left untreated or were stimulated with 100 nM PMA (Sigma) and 1 µM ionomycin (Calbiochem) for 30 min at 37°C, 5% CO2. Live/dead cell staining and anti-CD4 clone GK1.5 surface staining was performed as indicated followed by an intracellular FACS with anti-Foxp3 clone FJK-16s (eBioscience) and anti-phospho-NFKBp65 (Ser536) (93H1, Cell Signaling) antibodies. Following two washes with Perm/Wash buffer, cells were stained with an APC-labelled anti-rabbit IgG antibody (A10931, Invitrogen). For cytokine staining, cells were also stimulated with 100 nM PMA (Sigma) and 1 µM ionomycin (Calbiochem) for 4 h at 37°C, 5% CO2 in the presence of 1X Brefeldin A (BioLegend). Following live/dead cell and surface staining, cells were fixed and permeabilized using the Foxp3 staining kit (ebioscience).

Instrument

Sorting: FACSAriaTM III, otherwise FACSCantoTM II (BD Biosciences)

Software

FlowJo version 9.7.7 and 10.1r7; Ideas 6.2.183.0 (Merck) and FCSExpress6Plus for Amnis Image Stream

Cell population abundance

Reanalysis of post-sort fractions:
CD4+EYFP+CD44loCD62Lhi naive Tregs >95%
CD4+CD25+CD45RBlo Tregs and CD4+CD25-CD45RBhi naive conventional T cells >96%

Gating strategy

Gate 1: FSC-A/SSC-A parameters were used for gating on lymphocytes
Gate 2: FSC-H/FSC-W parameters were used for gating on singlets.
Gate 3: SSC-H/SSC-W parameters were used for gating on singlets.
Gate 4: AmCyan-A or V450-A negative cells were used for gating on living cells

☒ Tick this box to confirm that a figure exemplifying the gating strategy is provided in the Supplementary Information.
